# Effects of public financing of essential maternal and child health interventions across wealth quintiles in Nigeria: an extended cost-effectiveness analysis

**DOI:** 10.1016/S2214-109X(23)00056-6

**Published:** 2023-03-14

**Authors:** Wenhui Mao, David Watkins, Miriam L Sabin, Katy Huang, Etienne Langlois, Yewande Ogundeji, Helga Fogstad, Marco Schäferhoff, Gavin Yamey, Osondu Ogbuoji

**Affiliations:** a Center for Policy Impact in Global Health, Duke Global Health Institute, Durham, NC, USA; b Margolis Center for Health Policy, Duke University, Durham, NC, USA; c Seattle, WA, USA; d The Partnership for Maternal, Newborn and Child Health, WHO, Geneva, Switzerland; e Health Strategy and Delivery Foundation, Maitama, Abuja, Nigeria; f Open Consultants, Berlin, Germany

## Abstract

**Background:**

Maternal and newborn mortality rates in Nigeria are among the highest globally, and large socioeconomic inequalities exist in access to maternal, newborn, and child health (MNCH) services in the country. Inequalities also exist in catastrophic health expenditure among households in Nigeria. We aimed to estimate the health and financial risk protection benefits across different wealth groups in Nigeria if a policy of public financing of MNCH interventions were to be introduced.

**Methods:**

We did an extended cost-effectiveness analysis to estimate the health and financial risk protection benefits, across different household wealth quintiles, of a public-financing policy that assumes zero out-of-pocket costs to patients at the point of care for 18 essential MNCH services. We projected health outcomes (deaths in children aged <5 years [under-5 deaths] and maternal deaths) and private expenditure averted using the Lives Saved Tool with data extracted from national surveys. We modelled three scenarios: 1) coverage expansion at a rate equal to the trend observed between 2013 and 2018 (status quo); 2) annual coverage expansion by 5% compared with the status quo (uniform scale-up scenario); and 3) annual coverage expansion by 10%, 8%, 6%, 4%, and 2% compared with the status quo from the poorest to the wealthiest quintiles, respectively (pro-poor scale-up scenario).

**Findings:**

Our analysis shows that, if an additional 5% increase in coverage was provided for all wealth quintiles between 2019 and 2030, this uniform scale-up policy would prevent more than 0·11 million maternal deaths and 1·05 million under-5 deaths, avert US$1·8 billion in private expenditure, and avert 3266 cases of catastrophic health expenditure. The incremental cost effectiveness ratio would be $44 per life-year gained, which is highly cost-effective when compared with the gross domestic product per capita of Nigeria for 2018 ($2028). The policy would prevent a higher number of under-5 deaths and catastrophic health expenditure cases in poorer quintiles, but would prevent more maternal deaths and private expenditure in wealthier quintiles. If poorer populations experienced a greater increase in service coverage (ie, the pro-poor scale-up scenario), more maternal and under-5 deaths would be prevented in the poorer quintiles and more private expenditure would be averted than would be under previous scenarios.

**Interpretation:**

Public financing of essential MNCH interventions in Nigeria would provide substantial health and financial risk protection benefits to Nigerian households. These benefits would accrue preferentially to the poorest quintiles and would contribute towards reduction of health and socioeconomic inequalities in Nigeria. The distribution would be more pro-poor if public financing of MNCH interventions could target poor households.

**Funding:**

WHO Partnership for Maternal, Newborn, and Child Health.

## Introduction

Despite global progress in improving maternal, newborn, and child health (MNCH), mortality remains high in low-resource settings. In 2019, 53% of global deaths in children younger than 5 years (hereafter referred to as under-5 deaths or under-5 mortality) occurred in sub-Saharan Africa.^1^ In Nigeria, maternal and newborn mortality rates are among the highest in the world.^2^ In 2018, 80% of married women were at high risk of complications during childbirth and the under-5 mortality rate was 132 deaths per 1000 livebirths.^3^ Furthermore, large socioeconomic inequalities exist in MNCH in Nigeria.^4^ For example, in the 2018 Demographic and Health Survey, skilled assistance at delivery was documented for 12% of deliveries in the poorest wealth quintile compared with 87% in the wealthiest quintile.^5^ If such unequal distribution of service use continues, poor families in Nigeria will continue to lag behind in access to essential MNCH services and in the attainment of the UN Sustainable Development Goals (SDGs).

Considering these inequalities, efforts have been initiated to improve the access to, and affordability of, health services in Nigeria, including the Free Maternal and Child Health Program implemented in 12 states,^5^ the National Health Insurance Scheme, and State Health Insurance Schemes.^6^ However, service implementation varies across different states and population groups. In 2017, out-of-pocket payments accounted for more than 77% of total health expenditure in Nigeria, and the incidence of catastrophic health expenses, defined as spending more than 10% of household expenses on health care, almost doubled between 2010 and 2016.^7^, ^8^ The Nigerian Government allocated only 4·6% of total expenditure to health in 2017, which is lower than the 15% target of the Abuja Declaration.^9^ However, the unstable price of oil and the COVID-19 pandemic have had an unpredictable effect on economic growth in the country, making it even more difficult to mobilise and allocate additional resources for health in the country.^8^ Thus, it is an important and urgent policy priority for the Nigerian Government to understand how to prioritise and allocate scarce domestic resources for health to achieve maximum benefits for households.

**Research in context**

**Evidence before this study**

We reviewed documents from WHO, Partnership for Maternal, Newborn, and Child Health (PMNCH), and UNICEF and searched PubMed for articles published in English until January, 2019, using the search terms “public financing”, “MNCH” and “economic evaluation”. Our search yielded six reports of high-quality research and global estimates about the health outcomes of different maternal, newborn, and child health (MNCH) interventions. We also identified economic evaluations of public financing of MNCH services in Ethiopia, India, and Malaysia. Existing studies showed that public financing of MNCH services could save lives and provide financial risk protection. However, these studies addressed one MNCH intervention or focused on one target population only.

**Added value of this study**

We estimated the health and financial risk protection benefits across different wealth quintiles if a policy of public financing of MNCH interventions were to be introduced in Nigeria. Our study also addressed a package of 18 MNCH interventions and two key target groups: children younger than 5 years and pregnant women. Our findings contribute to the ongoing discussions about the achievement of the Sustainable Development Goals in Nigeria related to poverty, maternal and child mortality, universal health coverage, and gender equality.

**Implications of all the available evidence**

Our study found that if a policy of public financing of MNCH interventions were to be introduced, it would save lives, prevent household catastrophic health expenditure, would be highly cost-effective, and would preferentially benefit poorer populations (pro-poor). Additionally, the annual cost of such interventions would be less than 10% of current domestic government spending on health in Nigeria. Our findings imply that public financing of essential MNCH interventions in Nigeria would provide substantial health and financial risk protection benefits to Nigerian households. These benefits would accrue preferentially among individuals in the poorest quintiles and would contribute towards the reduction of health and socioeconomic inequalities in Nigeria. Furthermore, the distribution of health and financial benefits would be more pro-poor if public financing of MNCH interventions targeted poor households.

We did an extended cost-effectiveness analysis to estimate the health and financial risk protection benefits across different wealth groups if a policy of public financing of MNCH interventions were to be introduced. Such policy is aligned with the WHO universal health coverage objectives to eliminate user fees at the point of care and improve financial risk protection. Although the costs and health benefits of MNCH interventions have been well reported, few studies have reported the distribution of benefits across wealth groups and few have focused on financial risk protection.

## Methods

### Study setting and assumptions

We did an extended cost-effectiveness analysis of a policy to publicly finance essential MNCH interventions in Nigeria.^10^ Compared with traditional cost-effectiveness analyses that estimate the levels of cost and effectiveness of certain interventions, an extended cost-effectiveness analysis further estimates: 1) the distribution of the health benefits across a spectrum; 2) the private health-care expenditure averted by the policy; and 3) the financial risk protection benefits that the policy provides. Under this policy, MNCH interventions are provided to the patient without any cost at the point of care (ie, no out-of-pocket payments). The projection period was between 2019 and 2030 and costs were reported in US$ (whereby $1 was equal to 305·79 Nigerian Naira; 2018 Central Bank of Nigeria rates). Wealth quintiles were defined at the beginning of the projection period and we assumed that an individual's wealth quintile remained stable throughout the projection period. All analysis and projections were conducted using the Lives Saved Tool (version 4.761) and results were reported according to the Consolidated Health Economic Evaluation Reporting Standards checklist. The Lives Saved Tool is a linear, deterministic mathematical model; further details are in the appendix (p 28). The research protocol was reviewed and approved by Duke Campus Institutional Review Board (2020-0122).

### MNCH package definition

We selected interventions that met the following inclusion criteria: 1) recommended as priority MNCH interventions by the Partnership for Maternal, Newborn and Child Health (PMNCH),^11^ an international multi-stakeholder alliance, including governments, UN agencies, health-care professional associations, youth-led organisations, and non-governmental organisations; 2) data on coverage for these MNCH interventions available by wealth quintiles; 3) interventions not currently provided for free in Nigeria; and 4) interventions included in the list of Lives Saved Tool's default interventions. We included 18 interventions (panel; appendix p 3).

Panel **Interventions and packages included in the extended cost-effectiveness analysis**

• Tetanus toxoid vaccination* †
• Intermittent preventive treatment of malaria during pregnancy*†
• Iron supplementation in pregnancy*
• Hypertensive disorder case management in pregnancy*
• Malaria case management in pregnancy*
• Childbirth package including the following six interventions: labour and delivery management (including clean birth practices); antibiotics for pre-term premature rupture of the membranes; MgSO_4_ for the management of eclampsia; active management of the third stage of labour; immediate assessment and stimulation of neonate; and neonatal resuscitation*†
• Promotion of breastfeeding†
• Chlorhexidine cleaning of umbilical cord†
• Complementary feeding (education only)†
• Complementary feeding (supplementary feeding and education)†
• Vitamin A supplementation†
• Household protection from malaria (insecticide-treated bednets and indoor residual spraying)*†
• Provision of oral rehydration solution†
• Zinc for treatment of diarrhoea†
• Oral antibiotics for pneumonia†
• Artemisinin compounds for treatment of malaria†
• Treatment for severe acute malnutrition†
• Treatment for moderate acute malnutrition†

Excluded interventions and reasons for exclusion are in the appendix (p 3).

### Baseline data and sources

We divided the total 2018 population of Nigeria (191 million)^12^ into five quintiles based on household wealth using 2018 Nigeria Demographic and Household Survey data.^13^ For each wealth quintile, we obtained the quintile-specific baseline disease prevalence and service coverage from 2018 Nigeria Demographic and Health Surveys and Nigeria Multiple Indicator Cluster Surveys,^4^, ^13^ and estimated the average annual rates of change (AARC) for each quintile using the quintile-specific data from the previous two surveys (appendix p 5). Proxies for service coverage were used when no reliable data were available (appendix p 6). We adjusted the default settings of the Lives Saved Tool for target population and population in need by using quintile-specific estimates for total fertility rates, proportion of women with low BMI (<18·5 kg/m^2^), neonatal mortality rates, infant mortality rates, and under-5 mortality rates (appendix p 7).

### Modelled scenarios

We modelled three scenarios: 1) coverage for interventions will expand at an AARC equal to the trend observed between 2013 and 2018 (referred to hereafter as status quo; appendix p 9); 2) coverage will increase by 5% compared with the status quo scenario every year^14^ (referred to hereafter as the uniform scale-up scenario); and 3) coverage will increase by 10%, 8%, 6%, 4%, and 2% compared with the status quo from the poorest to the wealthiest household quintiles, respectively, every year (referred to hereafter as the pro-poor targeted scale-up scenario). For all three scenarios, if coverage reached 95%, it would remain stable until the end of the projection (appendix pp 9–21).

### Estimation of outcomes

Health outcomes were the number of under-5 deaths and maternal deaths averted and life-years gained. We modelled deaths averted using the Lives Saved Tool. To estimate life-years saved, for under-5 deaths, we multiplied the number of deaths averted at age 5 by the remaining life expectancy at age 5, and for maternal deaths (ie, among women of reproductive age), we multiplied the number of deaths averted at 30 years (midpoint between 15 and 45 years) by the remaining life expectancy at 30 years.^10^

The Lives Saved Tool estimates costs on the basis of a target population, proportion of population in need, coverage, treatment inputs, and cost per service. The Lives Saved Tool methodology has been published previously (appendix p 29).^15^, ^16^ We reported the intervention cost from the Lives Saved Tool, which included health-care provider fees, diagnostic costs, and medication costs, but excluded indirect costs such as transportation costs or the opportunity cost of lost employment or wages (appendix p 8). We applied a scale-up cost of 20% to estimate the total health system cost of the policy.

Private expenditure refers to pooled resources that are not controlled by the government, such as voluntary health insurance, and direct payments or out-of-pocket payments from households. In Nigeria, voluntary health insurance accounted for only 0·55% of current health expenditure in 2018.^9^ Therefore, to estimate the private expenditure averted, we used the total cost of interventions from the Lives Saved Tool, and multiplied the cost by the current out of-pocket payment ratio in Nigeria (77%). We defined catastrophic health expenditure as private expenditure for health expenditures that exceeded 10% of household income.^17^ We used the private expenditure for each intervention to estimate whether this intervention could lead to catastrophic health expenditure and accumulated the number of catastrophic health expenditure cases for each intervention. We used the income information from the Nigeria Living Standards Survey, disaggregated by wealth quintiles^18^ (appendix p 8). Incremental cost-effectiveness ratios were reported from a modified societal perspective that includes the perspective of the payer and the household.

We conducted a sensitivity analysis of the effect of the total fertility rate on estimates, to assess the effect of quintile population size and population growth rate. We repeated our analysis with different total fertility rate assumptions (using fertility rates 10% lower and higher than estimated fertility rate and using the national mean total fertility rates for each quintile). We also conducted sensitivity analyses on discount rates (at 3%, 5% and 10%), scale-up costs (at 10%, 30%, and 40%), and service coverage levels (at 4% and 6%).

### Role of the funding source

The study funder was involved in study design, but had no role in data collection, data analysis, data interpretation, or writing of the report.

## Results

Our model estimates a total number of under-5 deaths over the 12-year period of 11·5 million in the status quo scenario, 10·4 million in the uniform scale-up scenario, and 10·1 million in the pro-poor scale-up scenario. Over the same period, we estimate that maternal deaths would be 0·79 million in the status quo scenario, 0·68 million in the uniform scale-up scenario, and 0·67 million in the pro-poor scale-up scenario (table 1). Compared with the status quo scenario, 1·05 million under-5 deaths would be averted under a uniform scale-up scenario, resulting in 57·92 million life-years gained between 2019 and 2030 (table 2). The distribution of averted under-5 deaths would benefit poorer populations, with 45·5% of total deaths averted in the poorest two quintiles. Compared with the status quo scenario, under the pro-poor targeted scale-up scenario, 60·3% of all under-5 deaths averted would be in the poorest two quintiles (table 2; appendix p 15).

**Table 1:** Numbers of under-5 and maternal deaths for each scenario and annual rate of change in Nigeria, disaggregated by household wealth quintile (2019–30)

		**Baseline (2018)**	Total deaths, n*		
			Scenario 1: status quo	Scenario 2: uniform scale-up	Scenario 3: pro-poor targeted scale-up
**Under-5 deaths**					
Household wealth quintile†					
	1 (poorest)	270 811	3 632 624	3 405 555	3 205 237
	2	257 041	3 314 364	3 063 562	2 923 369
	3	186 531	2 338 980	2 082 600	2 034 518
	4	124 704	1 553 794	1 344 033	1 380 135
	5 (wealthiest)	60 580	652 194	546 717	592 215
National		899 667	11 491 956	10 442 467	10 135 474
**Maternal deaths**					
Household wealth quintile†					
	1 (poorest)	14 572	191 126	181 671	169 931
	2	13 484	171 559	155 607	147 306
	3	12 179	157 221	131 648	126 705
	4	12 179	158 499	127 438	133 744
	5 (wealthiest)	8265	111 252	82 029	94 245
National		60 679	789 657	678 393	671 931

See panel for details of which interventions applied to under-5 deaths and which applied to maternal deaths.

* 2019–30.

† Quintiles were defined at the beginning of the analysis period (2018) and the population was considered as a cohort between 2018 and 2030—ie, individuals maintained their quintiles during the period of interest (2018–30).

**Table 2:** Additional under-5 and maternal deaths averted through public financing of 18 essential maternal, newborn, and child health interventions in Nigeria, disaggregated by household wealth quintile (2019–30)

			Number of deaths averted*				**Proportion of total deaths averted, %**	Life-years gained*			
				2020	2025	2030	Total (2019–30)	2020	2025	2030	Total (2019–30)
**Uniform scale-up scenario**†											
Under-5 deaths											
	Household wealth quintile‡										
		1 (poorest)	5956	18 087	37 821	227 069	21·6%	328 712	998 222	2 087 341	12 531 938
		2	5877	19 845	45 289	250 802	23·9%	324 352	1 095 246	2 499 500	13 841 762
		3	5624	23 839	37 161	256 380	24·4%	310 389	1 315 674	2 050 916	14 149 612
		4	5247	19 102	28 691	209 761	20·0%	289 582	1 054 239	1 583 456	11 576 710
		5 (wealthiest)	3236	10 468	11 552	105 477	10·1%	178 595	577 729	637 555	5 821 276
	National		25 940	91 341	160 514	1 049 489	NA	1 431 629	5 041 110	8 858 768	57 921 298
Maternal deaths											
	Household wealth quintile‡										
		1 (poorest)	185	595	1958	9455	8·5%	6854	22 045	72 544	350 308
		2	219	1527	2695	15 952	14·3%	8,114	56 575	99 850	591 022
		3	368	2516	3447	25 573	23·0%	13 634	93 218	127 711	947 480
		4	692	2399	4607	31 061	27·9%	25 639	88 883	170 689	1 150 810
		5 (wealthiest)	613	2985	3265	29 223	26·3%	22 712	110 594	120 968	1 082 712
	National		2077	10 022	15 972	111 264	NA	76 953	371 315	591 763	4 122 331
**Pro-poor targeted scale-up scenario**†											
Under-5 deaths											
	Household wealth quintile‡										
		1 (poorest)	11 511	39 881	68 323	427 387	31·5%	635 292	2 201 032	3 770 746	23 587 489
		2	9435	32 094	63 655	390 995	28·8%	520 718	1 771 268	3 513 119	21 579 014
		3	6655	27 495	47 842	304 462	22·4%	367 289	1 517 449	2 640 400	16 803 258
		4	4274	15 799	27 334	173 659	12·8%	235 882	871 947	1 508 563	9 584 240
		5 (wealthiest)	1353	4333	9451	59 979	4·4%	74 672	239 138	521 601	3 310 241
	National		33 228	119 602	216 605	1 356 482	NA	1 833 853	6 600 834	11 954 430	74 864 242
Maternal deaths											
	Household wealth quintile‡										
		1 (poorest)	364	1920	4522	21 195	18·0%	13 486	71 136	167 540	785 275
		2	355	1918	3935	24 253	20·6%	13 153	71 062	145 792	898 574
		3	441	2768	5045	30 516	25·9%	16 339	102 554	186 917	1 130 618
		4	553	2020	4 609	24 755	21·0%	20 489	74 841	170 763	917 173
		5 (wealthiest)	271	888	3171	17 007	14·4%	10 041	32 900	117 486	630 109
	National		1984	9514	21 282	117 726	NA	73 507	352 494	788 498	4 361 748

See panel for details of which interventions applied to under-5 deaths and which applied to maternal deaths. NA=not applicable.

* Number of deaths averted and life-years gained were not discounted; to estimate life-years saved, we multiplied number of deaths averted at age *x* by the remaining life expectancy at age *x*, where *x* was 5 years for children and 30 years (midpoint between 15 and 45 years) for women of reproductive age; we used 55·19 years as the remaining life expectancy for children and 37·05 years for women.

† Estimates represent the difference between the specified scenario and the status quo scenario; for each scenario, additional lives were estimated as the difference between the number of deaths in the index year and the number of deaths in the baseline year (2018).

‡ Quintiles were defined at the beginning of the analysis period (2018) and the population was considered as a cohort between 2018 and 2030—ie, individuals maintained their quintiles during the period of interest (2018–30).

0·11 million maternal deaths could be averted through the uniform scale-up scenario, resulting in 4·12 million life-years gained between 2019 and 2030 (table 2). However, the distribution of averted maternal deaths would not be pro-poor under the uniform scale-up scenario: the two poorest quintiles would account for only 22·8% of averted deaths, whereas 54·2% of all maternal deaths averted would be among the wealthiest two quintiles. Compared with the status quo scenario, 38·6% of total maternal deaths would be averted among the two poorest quintiles under the pro-poor scale-up scenario, with a higher proportion of maternal deaths averted than in the status quo scenario (table 2; appendix p 15).

Different interventions would have different effects on the number of under-5 deaths averted overall and by wealth quintile (appendix pp 9, 15). Skilled birth assistance at delivery would prevent the most under-5 deaths (350 000), followed by provision of oral rehydration solution (252 000).

Overall, compared with the status quo scenario, the uniform scale-up scenario would save $1·77 billion in private expenditure between 2019 and 2030 (table 3). The largest savings in private expenditure costs would occur among the middle and wealthier quintiles (quintiles 3 and 4; 48·3%) between 2019 and 2030, while the smallest savings in private expenditure would be in the wealthiest quintile (14·1%). The pro-poor scenario would also have a pro-poor distribution in private expenditures averted, with the largest savings in private expenditure in the two poorest quintiles (55·5%) between 2019 and 2030. Private expenditures averted from skilled birth assistance at delivery would result in the largest savings in total private expenditure between 2019 and 2030, followed by savings from zinc for treatment of diarrhoea and oral rehydration solution for diarrhoea (appendix p 17). The total health system cost as a result of the scenarios between 2019 and 2030 would be $2·76 billion through the uniform scale-up scenario and $3·51 billion through the pro-poor scenario (appendix pp 14, 20).

**Table 3:** Private expenditure averted from public financing of 18 essential maternal, newborn, and child health interventions in Nigeria, disaggregated by income quintile (2019–30)

		**2020**	**2025**	**2030**	**Total (2019–30)**	**Proportion of total private expenditure averted, %**
Uniform scale-up scenario*						
Household wealth quintile†						
	1 (poorest)	4·76	23·94	63·38	314·78	17·8%
	2	5·33	27·08	71·78	350·00	19·8%
	3	7·20	36·25	69·83	425·07	24·0%
	4	9·15	42·37	58·64	429·50	24·3%
	5 (wealthiest)	8·39	23·84	30·31	248·68	14·1%
National		34·82	153·47	293·93	1768·03	NA
Pro-poor targeted scale-up scenario*						
Household wealth quintile†						
	1 (poorest)	9·57	54·75	127·16	663·48	29·4%
	2	8·62	45·77	112·50	588·16	26·1%
	3	8·60	43·68	78·20	492·63	21·8%
	4	7·29	35·63	54·72	370·24	16·4%
	5 (wealthiest)	3·36	13·23	19·90	140·91	6·3%
National		37·43	193·06	392·48	2255·41	NA

See panel for details of which interventions applied to under-5 deaths and which applied to maternal deaths. Data are US$ million (2018 Central Bank of Nigeria rates), unless otherwise stated. NA=not applicable.

* Estimates represent the difference between the specified scenario and the status quo scenario; for each scenario, private expenditure averted was estimated as the difference between the estimated private expenditure in the index year and the estimated private expenditure in the baseline year (2018).

† Quintiles were defined at the beginning of the analysis period (2018) and the population was considered as a cohort between 2018 and 2030—ie, individuals maintained their quintiles during the period of interest (2018–30).

In 2020, under the uniform scale-up scenario, financial risk protection provided through public financing of MNCH interventions would prevent catastrophic health expenditure among 3266 individuals (table 4). Under the pro-poor targeted scale-up scenario, more than 62% of cases of catastrophic health expenditure would be averted among the poorest quintile, while 59% of cases would be averted under the uniform scale up scenario (table 4).

**Table 4:** Cases of catastrophic health expenditure averted by public financing of 18 maternal, newborn, and child health interventions in Nigeria, by income quintile (2019–30)

		**Cases of catastrophic health expenditure spending averted, n**	Proportion of total catastrophic health expenditure averted, %*
**Uniform scale-up scenario**†			
Household wealth quintile‡			
	1 (poorest)	1640	50·2%
	2	771	23·6%
	3	561	17·2%
	4	294	9·0%
	5 (wealthiest)	0	0
National		3266	NA
**Pro-poor targeted scale-up scenario**†			
Household wealth quintile‡			
	1 (poorest)	3336	62·1%
	2	1237	23·0%
	3	561	10·4%
	4	236	4·4%
	5 (wealthiest)	0	0
National		5369	NA

NA=not applicable.

* Catastrophic health expenditure was defined as out-of-pocket payment for health expenditure that exceeded 10% of household income.

† Estimates represent the difference of the specified scenario and the status quo scenario; for each scenario, cases of catastrophic health expenditure were estimated as the difference between the number of deaths in the index year and the number of deaths in the baseline year (2018).

‡ Quintiles were defined at the beginning of the analysis period (2018) and the population was considered as a cohort between 2018 and 2030—ie, individuals maintained their quintiles during the period of interest (2018–30).

Compared with the status quo scenario, the incremental cost per life saved through the uniform scale-up scenario between 2019 and 2030 would be $2374, while the cost per life-year saved would be $44 in the same period (table 5). Compared with the status quo scenario, the incremental cost-effectiveness ratio (ICER) under the pro-poor scale-up scenario would be $2384 per life saved. Our results suggest that the policy to publicly finance MNCH interventions would be more cost-effective in poorer quintiles than in wealthier quintiles. Chlorhexidine would be the most cost-effective intervention with the lowest ICER for children younger than 5 years and case management for hypertensive disorders in pregnancy would be most cost-efficient for pregnant women. By contrast, supplementary feeding and education would be the least cost-effective interventions for children younger than 5 years and iron supplementation in pregnancy for pregnant women (appendix p 13).

**Table 5:** Incremental cost-effectiveness ratios from public financing of 18 maternal, newborn, and child health interventions in Nigeria, disaggregated by income quintile (2019–30)

		Incremental cost per life saved, US$*				Incremental cost per life-year saved, US$*			
		2020	2025	2030	Mean (SD), 2019–30	2020	2025	2030	Mean (SD), 2019–30
**Uniform scale-up scenario**									
Household wealth quintile†									
	1 (poorest)	1208	1997	2483	2074 (431)	22·1	36·6	45·7	38·1 (8·0)
	2	1362	1974	2331	2045 (373)	25·0	36·6	43·0	37·8 (6·9)
	3	1872	2143	2680	2349 (417)	34·6	40·1	49·9	43·9 (7·8)
	4	2402	3071	2744	2779 (283)	45·3	57·8	52·1	52·6 (5·3)
	5 (wealthiest)	3397	2762	3188	2877 (462)	64·9	54·0	62·3	56·1 (8·7)
National		1937	2360	2596	2374 (278)	36·0	44·2	48·5	44·4 (5·2)
**Pro-poor targeted scale-up scenario**									
Household wealth quintile†									
	1 (poorest)	1255	2041	2720	2305 (528)	23·0	37·6	50·3	42·4 (9·8)
	2	1373	2097	2594	2207 (431)	25·2	38·7	47·9	40·8 (8·0)
	3	1888	2249	2304	2292 (398)	34·9	42·0	43·1	42·8 (7·4)
	4	2354	3117	2669	2908 (382)	44·3	58·7	50·8	54·9 (7·2)
	5 (wealthiest)	3222	3949	2457	2852 (674)	61·8	75·8	48·5	55·7 (12·4)
National		1657	2330	2571	2384 (383)	30·6	43·3	48·0	44·4 (7·2)

* Estimates were not discounted; to estimate life-years saved, we multiplied number of deaths averted at age *x* by the remaining life expectancy at age *x*, where *x* was five years for children and 30 years (midpoint between 15 and 45 years) for women of reproductive age.

† Quintiles were defined at the beginning of the analysis period (2018) and the population was considered as a cohort between 2018 and 2030—ie, individuals maintained their quintiles during the period of interest (2018–30).

Sensitivity analysis found that when the total fertility rate increased by 10%, the national estimates of deaths averted and private expenditure averted increased by around 9·6% and when total fertility rate decreased by 10% estimates of deaths averted and private expenditure averted decreased by around 9·6%, with little variation across quintiles. ICERs would remain almost the same (appendix p 24). If we assume that all quintiles have the national average total fertility rate, the total number of deaths averted would decrease by 5·1% and private expenditure by 4·5%, with substantial variation among the different quintiles and the ICER would increase by 0·6% (appendix p 24). Sensitivity analysis of discount rates found that if a 3% discount was applied, the private expenditure averted and the ICER would increase by 30·9% with little variation across quintiles. The ICER for discount rates at 3%, 5% and 10% would be $3071, $3645, and $5573 per life-year saved, respectively (appendix p 25). Sensitivity analysis of scale-up costs found that if scale-up cost increased or decreased by 10%, it would lead to about 8·3% increase or decrease in ICER, respectively, while total lives saved and private expenditure averted would remain stable (appendix p 26). If scale-up rate was 4% per year, 15·1% fewer deaths would be averted and there would be a 15·6% decrease in private expenditure averted. By contrast, if scale-up rate was 6% per year, the total number of deaths averted would increase by 19·3% and private expenditure averted would increase by 16·5% (appendix p 27).

## Discussion

Our study found that a policy to provide public financing for priority MNCH interventions in Nigeria has the potential to save lives and prevent catastrophic health expenditure, would be highly cost-effective, and would have a pro-poor distribution.

Our results support findings from other evaluations of public financing of MNCH services in resource-poor settings. For example, Nandi and colleagues found that scaling up of a home-based neonatal care package in rural India through public financing would prevent five deaths and provide $285 of insurance per 1000 livebirths, with most benefit for poor families.^19^ Other studies found public financing for rotavirus vaccinations would decrease deaths and impoverishment in poorer quintiles in Ethiopia and India,^20^, ^21^ and in Malaysia it would provide financial risk protection across all quintiles.^22^ Although the findings of these studies strengthen our findings, each study addressed a single MNCH intervention or focused on one target population. By contrast, our study addresses a package of MNCH interventions and two key target groups—children younger than 5 years and pregnant women.

Additionally, our study focuses on Nigeria, a country that accounts for a substantial proportion of the global burden of maternal mortality,^23^, ^24^ under-5 mortality,^25^ and poverty.^26^ Our findings suggest that investment in public financing for MNCH could reduce mortality and alleviate poverty, providing an important contribution to ongoing policy discussions about the best approaches for achieving the SDG goals for poverty, health, and gender equality in Nigeria, and as the *Lancet* Nigeria Commission suggested, prioritisation of health could be the first place to start.^27^

It is important that decision makers explore policies that provide both health and financial benefits to women and children who are the most susceptible to the health and financial risks of seeking and of forgoing essential health care.^28^, ^29^ Our study estimated that the cost of publicly financing 18 MNCH interventions would be less than $2374 per death averted, or $44 per life-year gained, which is highly cost-effective when compared with the gross domestic product (GDP) per capita of Nigeria ($2027 in 2018; WHO recommends three times GDP as the threshold for cost-effectiveness^30^). We also found that most of the 18 MNCH interventions included in our study were pro-poor and that implementing a policy of publicly financing these interventions would improve access and reduce health disparities in Nigeria. The total health system cost of providing 18 interventions between 2019 and 2030 would be $2·76–3·51 billion, translating into a mean annual cost of $0·23–0·29 billion, equating to less than 10% of the current domestic government spending on health in Nigeria ($2·3 billion in 2019).

At present in Nigeria, coverage of health services is higher among wealthier quintiles, whereas the poorest quintile has the least coverage. Thus, there is an opportunity for considerable improvement in the service coverage for the poorest populations. Different findings from two scenarios suggested that if the policy could reach poor populations, better health outcomes and financial risk protection would be achieved. Additionally, the health benefits and financial risk protection would have a stronger pro-poor distribution. In reality, the pro-poor targeted scale-up scenario could be implemented by targeting populations in less developed areas, such as the rural regions or lowest-income districts or states. In 2019, the Basic Healthcare Provision Fund (BHCPF) was launched in Nigeria to provide universal health coverage to citizens. BHCPF currently prioritises people living in poor households through geographical targeting and coverage of services preferentially used by poorer populations. At primary health-care centres, BHCPF covers the cost of antenatal care, delivery, and postnatal care for pregnant women, pneumonia treatment, diarrhoea treatment, and malaria treatment for children younger than 5 years.^31^ BHCPF could therefore be the vehicle for the implementation of the pro-poor scenario. However, for policy implementation to be successful, problems that have affected previous free (at the point of care) maternal and child health programmes must be addressed. Onwujekwe and colleagues assessed one such free programme and identified corrupt practices, scarcity of medical supplies, and weak public financial management as barriers that prevented the programme from achieving the intended effect.^5^ The Nigerian Government committed $2·3 billion to the PMNCH Call to Action on COVID-19 for 2020–28 for strategic interventions, which could potentially improve access to MNCH services while reducing health inequalities.

Our study has several limitations. First, due to difficulties in obtaining disaggregated data by socioeconomic quintile for all MNCH interventions recommended by WHO, we only included a package of 18 MNCH interventions in this analysis. This relatively small number of MNCH interventions with reliable disaggregated data indicates the importance of bridging the data gap for MNCH interventions. Additionally, there might be gaps in implementation of interventions currently being offered for free and efforts to expand the scope of MNCH services, under considerable resource limitations, should be balanced against efforts to improve access and quality of services that are currently offered. Second, our study assigned wealth quintiles at baseline (in 2018) and quintiles were considered as cohorts for the period 2019 to 2030. Thus, we assumed that all individuals and families remained in the same wealth quintile for a period of 12 years, which might not be the case for some individuals or families. However, since we used relative socioeconomic measures rather than absolute measures, we controlled for the assumption that as absolute conditions (eg, wages) change for most members of society, relative conditions (compared with others) would on average remain the same. Third, our study focuses on MNCH interventions in Nigeria and might not be generalisable to other countries with different patterns of disease, health seeking behaviours, and socioeconomic characteristics. Fourth, the Lives Saved Tool is a deterministic model and we used the national estimate of population in need in the Lives Saved Tool for all quintiles, which might not reflect the real situation. We reported lives saved (deaths averted) but quality of life was not considered. Fifth, our study did not consider quality of care, effective coverage, or other factors that would affect service use such as direct non-medical costs (eg, cost of seeking care) and indirect costs (eg, lost wages), but we acknowledge the possibility that public financing of MNCH interventions might improve access or induce demand, but not necessarily improve the actual service use or quality.

In summary, this study shows that a policy to publicly finance priority MNCH interventions in Nigeria could bring additional health benefits and financial risk protection for all for an annual increase of 10% in the government health budget. Additionally, the distribution of the health benefits (under-5 and maternal deaths averted) and financial benefits (private expenditure averted and financial risk protection afforded) of this policy would be pro-poor. The distribution of health and financial benefits would be more pro-poor if public financing of MNCH interventions was targeted at poor households. Therefore, as Nigeria continues to deal with high maternal mortality, high under-5 mortality, and high levels of poverty, decision makers should strongly consider policies such as public financing for MNCH services that will result in progress towards achieving SDG targets for health, poverty, and gender equality. Protecting funding for these essential MNCH services should be prioritised to improve the lives of women and children and to reduce the inequalities in health and economic outcomes that exist in Nigeria.

## Data sharing

This study made use of publicly available data. The appendix describes the data sources and methods used in detail.

## Declaration of interests

MLS was an employee at the Partnership for Maternal, Newborn and Child Health (PMNCH) hosted by WHO during the execution of this research; she is currently an employee at *The Lancet*, but was recused from all editorial discussions about this manuscript. MS reports grants from PMNCH, Bill & Melinda Gates Foundation, Save the Children, Plan International, and WHO. OO reports grants from PMNCH during the conduct of the study; grants from the Gates Foundation, the National Institute of Minority Health and Health Disparities, WHO, and Gavi, the Vaccine Alliance; and personal fees from PMNCH and Save the Children outside the submitted work; and is a member of the Merck for Mothers Strengthening Systems for Safer Childbirth Advisory Committee and the Africa CDC Health Economics Unit Technical Advisory Group (both unpaid). All other authors declare no competing interests.

## Footnotes

* Intervention applied to maternal deaths.

† Intervention applied to under-5 deaths.
