# Supplementary material for: Effects of public financing of essential maternal and child health interventions across wealth quintiles in Nigeria: an extended cost-effectiveness analysis
Source: Lancet Glob Health. 2023 Mar 14;11(4):e597–605. doi: 10.1016/S2214-109X(23)00056-6 (PMC10030457; doi:10.1016/S2214-109X(23)00056-6)
Supplement: Supplementary appendix [file mmc1.pdf]

THE LANCET
Global Health

Supplementary appendix

This appendix formed part of the original submission and has been peer reviewed. We post it as supplied by the authors.

Supplement to: Mao W, Watkins D, Sabin ML, et al. Effects of public financing of essential maternal and child health interventions across wealth quintiles in Nigeria: an extended cost-effectiveness analysis. *Lancet Glob Health* 2023; **11:** e597–605.

Table of Contents

Section A. Selection of interventions ... ***3***

Table A1. Priority MNCH interventions included in this study ... 3

Section B. Parameters for projection ... ***5***

Table B1. Parameters for population and baseline health conditions for each wealth quintile ... 5

Table B2. Parameters for service coverage for each intervention, disaggregated by wealth quintile ... 6

Table B3. Intervention coverage for each intervention by 2030, disaggregated by wealth quintile ... 7

Section C. Estimation of financial risk protection ... ***8***

Table C1. Average household income by quintile and Individual equivalents ... 8

Section D. Additional tables and Figures ... ***9***

D1. Uniform scale up scenario ... 9

Table D1. Additional under-five and maternal deaths averted through public finance of 18 essential MNCH interventions in Nigeria, disaggregated by income quintile, and intervention. ... 9

Table D2. Additional private expenditure averted from public finance of selected priority MNCH interventions in Nigeria, disaggregated by income quintile, and intervention. ... 11

Table D3. Incremental cost-effectiveness ratios from public finance of MNCH interventions in Nigeria, disaggregated by income quintile, and intervention ... 13

Table D4. Cost of the public finance of 18 essential MNCH interventions in Nigeria, disaggregated by income quintile, and intervention ... 14

D2. Pro-poor targeted scale-up scenario ... 15

Table D5. Additional under-five and maternal deaths averted through public finance of 18 essential MNCH interventions in Nigeria, disaggregated by income quintile, and intervention. ... 15

Table D6. Additional private expenditure averted[1] from public finance of selected priority MNCH interventions in Nigeria, disaggregated by income quintile, and intervention. ... 17

Table D7. Incremental cost-effectiveness ratios from public finance of MNCH interventions in Nigeria, disaggregated by income quintile, and intervention ... 19

Table D8. Cost of the public finance of 18 essential MNCH interventions in Nigeria, disaggregated by income quintile, and intervention ... 20

D3. Concentration curves ... 21

Section E. Sensitivity analysis ... ***24***

Table E1. Summary for sensitivity analysis on TFR, disaggregated by socioeconomic quintiles ... 24

Table E2. Summary for sensitivity analysis on discount rate, disaggregated by socioeconomic quintiles ... 25

Table E3. Summary for sensitivity analysis on scale up cost, disaggregated by socioeconomic quintiles ... 26

Table E4. Summary for sensitivity analysis on service coverage, disaggregated by socioeconomic quintiles ... 27

Section F. LiST Methodologies ***28***
F1 Overview 28
F2. Structure 28
F3. Costing 29

Section A. Selection of interventions

The *Essential Interventions, Commodities and Guidelines for Reproductive, Maternal, Newborn and Child* Health[1] guide was used as a guide in selecting interventions for inclusion in this study. This list contains a package of 42 reproductive maternal and child health (MNCH) priority interventions for which there is consensus that there is strong evidence of effectiveness of the intervention, and of the delivery strategy. From this list of 42, we first excluded interventions for which there was no data (or data was unreliable) for different wealth quintiles, then we excluded interventions that are already provided for free in Nigeria (Routine Immunization Program [1]). Finally, the remaining interventions were mapped with the interventions contained in the Lives Saved Tool (LiST). Of the final list of 18 interventions included in the study, six were considered as a package of care called skilled birth assistance at delivery, while the others were modeled separately. See table A1 below for details.

Although these eighteen packages represent a subset of comprehensive essential MNCH interventions, they are illustrative of the potential effects of public financing of MNCH in Nigeria.

Table A1. Priority MNCH interventions included in this study

Priority Intervention	Target population	Source of data
1. Tetanus toxoid vaccination	Pregnant women	NDHS
2. Intermittent preventive treatment of malaria during pregnancy	Pregnant women	NDHS
3. Iron supplementation in pregnancy	Pregnant women	NDHS
4. Hypertensive disorder case management in pregnancy	Pregnant women	NDHS. Use "percentage of ANC visit from skilled provider * percentage of ANC visits with blood pressure measured" as proxy
5. Malaria case management in pregnancy	Pregnant women	NMICS. Use "percent of pregnant women age 15-49 in all households * percentage who slept under ITN last night" as proxy
6. Skilled birth assistance at delivery (includes the following):<br>1) Labor and delivery management (including clean birth practices)<br>2) Antibiotics for pre-term premature rupture of the membranes,<br>3) MgSO4 for the management of eclampsia<br>4) Active management of the third stage of labor<br>5) Immediate assessment and stimulation of newborn,<br>6) Neonatal resuscitation.	Pregnant women and newborns	NDHS
7. Breastfeeding promotion	Livebirth	NDHS. Use "percent of children 1-5 months of age that are exclusive breastfed" as proxy
8. Chlorhexidine	Livebirth	NDHS. Among recent live births in the 2 years preceding the survey, percentage with chlorhexidine applied to the stump of the umbilical cord. Since the provision of chlorhexidine was reported in 2018NDHS but not in 2013NDHS, we used the annual rate of change of skilled birth attendance as proxy
9. Complementary feeding-education only	Children 6-23 months	NDHS. Use "Percentage of women with a postnatal check" as proxy
10. Complementary feeding-supplementary feeding and education	Children 6-23 months	NDHS. Use "Percentage of women with a postnatal check" as proxy
11.Vitamin A supplementation	Children 6-59 months	NDHS

[1] Partnership for Maternal, Newborn and Child Health. (2011). Essential Interventions, Commodities and Guidelines for Reproductive, Maternal, Newborn and Child Health: A global review of the key interventions related to reproductive, maternal, newborn and child health.

12. Households protected from malaria (ITN+IRS)	Households	NMICS
13.Oral rehydration solution	Children 0-59 months	NDHS
14. Zinc for treatment of diarrhea	Children 0-59 months	NDHS
15. Oral antibiotics for pneumonia	Children 1-59 months	NDHS
16. Artemisinin-based combination treatment of malaria	Children 1-59 months	NDHS
17. SAM-treatment for severe acute malnutrition	Children 6-59 months	NDHS. Use "Minimal acceptable diet" as proxy
18. MAM-treatment for moderate acute malnutrition	Children 6-59 months	NDHS. Use "Minimal acceptable diet" as proxy

Reasons for exclusion

1) **No disaggregated data by wealth quintiles:**
- [PERICONCEPTUAL PACKAGE] Folic acid supplementation/fortification; Safe abortion services (also not legal in Nigeria); Post abortion case management; Ectopic pregnancy care.
- [PREGNANCY PACKAGE] Pregnancy case management; Blanket iron supplementation /fortification; Syphilis detection and treatment; Calcium supplementation; Multiple micronutrient supplementation in pregnancy; Balanced energy supplementation; Fetal growth restriction detection and management;
- [PREVENTIVE] Clean postnatal practices; Zinc supplementation; Improved sanitation -utilization of latrines or toilets; Improved water source; Hand washing with soap; Hygienic disposal of children's stools
- [CURATIVE] Maternal sepsis case management; Thermal care; KMC-kangaroo mother care; Full supportive care for prematurity; Case management of neonatal sepsis/pneumonia; Oral antibiotics for neonatal sepsis/pneumonia; Injectable antibiotics for neonatal sepsis/pneumonia; Full supportive care for neonatal sepsis/pneumonia Antibiosis for treatment of dysentery; Vitamin A for treatment of measles;

2) **Interventions already being provided for free in Nigeria:**
- [VACCINES] BCG-single dose; Polio-three doses; Pentavalent; DPT-three doses; H. influenzae type b- three doses; HepB-three doses; Pneumococcal-three doses; Rotavirus-two doses; Meningococcal A-single dose; Malaria vaccine-three doses; Measles-single dose

3) **Other reasons:**
- [PERICONCEPTUAL PACKAGE] Contraceptive use was excluded because the disaggregated data was not reliable.
- [PREGNANCY PACKAGE] Diabetes case management in pregnancy was excluded because LiST only model its impact on stillbirth, which is not the health impact assessed by this study

Section B. Parameters for projection

For each intervention, the target population was defined as the population that could potentially receive the intervention. Population in need was used to identify what share of the target population requires the intervention, per year. Population in need is determined by incidence and prevalence of conditions, as well as by treatment guidelines. Please visit https://avenirhealth.org/software-onehealth.php for more information related to LiST.

Using the baseline data, we adjusted several population-level parameters to reflect important differences between the wealth quintiles. We modified total fertility rates (TFR) according to 2018 DHS by wealth quintiles to project population growth to 2030. We also adjusted the proportion of women with low body mass index (BMI) (<18.5), neonatal mortality rate (NMR), infant mortality rate (IMR), and under five mortality rate (U5MR) by wealth quintiles based on 2018 NDHS (details can be found in Appendix section B, Table B1). These parameters show that poorer quintiles have a higher TFR, proportion of women with low BMI, NMR, IMR and U5MR.

Table B1. Parameters for population and baseline health conditions for each wealth quintile

Indicator	National	Quintiles: 1st (poorest)	2nd	3rd	4th	5th (wealthiest)	Source of data
Total Fertility Rate (%)[1]	5.3	6.7	6.2	5.6	5.6	3.8	2018 NDHS
Proportion of women with low BMI (<18.5) (%)	12.1	21.5	13.9	12.3	9.4	7.0	2018 NDHS
Neonatal mortality rate (per 1000 live births)	39	39	41	41	32	28	2018 NDHS
Infant Mortality Rate (per 1000)	67	78	77	69	53	40	2018 NDHS
Under 5 Mortality Rate(per 1000)	132	173	169	128	86	53	2018 NDHS

Notes

1. The Total Fertility Rate (TFR) is the number of live births a woman would have if she survived to age 50 and had children according to the prevailing pattern of childbearing at each age group

We obtained the actual coverage of each selected intervention in each wealth quintile from the most recent Nigeria Demographic Health Survey (NDHS) or Nigeria Multiple Indicator Cluster Surveys (MICS). (Please refer to section A, Table A for interventions and sources of data). We estimated the average annual rates of change (AARC) from the recent two surveys. In general, the poorer quintiles had lower coverage rate for MNCH interventions, but higher AARCs. The exception was ITN/IRS which had higher coverage among poorer quintiles (see Appendix section B, Table B2 for details). We set 95% as the highest coverage possible (see Appendix section B, Table B3 for details).

Table B2. Parameters for service coverage for each intervention, disaggregated by wealth quintile

			Actual service coverage (%)[3]						Average Annual Rate of Change (%)[4]					
				2018						Quintiles				
Interventions	Target population[1]	Population in need (%)[2]	National	1st (poorest)	2nd	3rd	4th	5th (wealthiest)	National	1st (poorest)	2nd	3rd	4th	5th (wealthiest)
1. Tetanus toxoid vaccination	Pregnant women	100	61.7	38.2	48.4	64.6	77.3	87.3	5	5	5	3	2	1
2. Intermittent preventive treatment of malaria during pregnancy	Pregnant women	100	16.6	12.1	10.9	15.9	22.8	24.1	5	5	5	5	5	5
3. Iron supplementation in pregnancy	Pregnant women	100	30.5	16.9	21.8	34.7	40.7	42.8	5	5	5	5	5	0
4. Hypertensive disorder case management in pregnancy	Pregnant women	1.1	62.9	36.5	48.6	67.9	80.3	90.4	3	5	5	2	0	0
5. Malaria case management in pregnancy	Pregnant women	24.8	58.0	67.8	70.2	59.1	43.0	38.0	5	5	5	5	5	5
6. Skilled birth assistance at delivery[5]	Pregnant women and newborns	100	43.4	13.4	23.6	45.4	65.9	84.3	3	5	5	3	1	0
7. Breastfeeding promotion	Livebirths	100	39.4	11.6	21.1	40.3	59.2	79.5	2	5	4	2	1	0
8. Chlorhexidine	Livebirths	100	32.8	28.1	31.3	36.0	36.1	33.8	5	5	5	5	5	5
9. Complementary feeding-education only	Children 6-23 months	46.5	10.9	5.2	7.2	11.2	14.0	19.6	3	5	5	3	1	0
10. Complementary feeding-supplementary feeding and education	Children 6-23 months	53.5	44.0	19.7	22.6	44.2	63.4	78.2	1	5	0	0	1	0
11. Vitamin A supplementation	Children 6-59 months	100	45.3	33.50	31.5	42.3	57.5	68.0	2	5	0	0	2	0
12. Households protected from malaria (ITN+IRS)	Households	99	60.6	73.2	70.4	62.1	54.2	47.8	4	5	5	3	3	2
13. Oral rehydration solution	Children 0-59 months	290.4	40.0	33.4	36.0	40.9	47.6	63.1	3	5	4	1	2	4
14. Zinc for treatment of diarrhea	Children 0-59 months	330	31.1	25.9	25.2	36.1	38.7	45.2	5	5	5	5	5	5
15. Oral antibiotics for pneumonia	Children 1-59 months	127.1	14.2	11.3	10.3	15.0	18.7	24.3	2	3	5	0	0	0
16. Artemisinin-based combination treatment of malaria	Children 1-59 months	59.4	7.6	3.6	5.4	7.0	10.1	17.0	5	0	5	3	4	5
17. SAM-treatment for severe acute malnutrition	Children 6-59 months	3.0	10.6	7.5	8.0	8.1	12.6	18.8	1	5	1	0	0	5
18. MAM-treatment for moderate acute malnutrition	Children 6-59 months	11.9	10.6	7.5	8.0	8.1	12.6	18.8	1	5	1	0	0	5

Notes

1. Target population is defined as the population that could receive and would benefit from the intervention. The default settings in LiST were used.
2. Population in need describes what share of the target population requires the intervention, per year. For most preventive care interventions, the share will be 100%. Population in need is determined by incidence and prevalence of conditions, as well as by treatment guidelines. The default settings in LiST were used.
3. Actual service coverage was extracted from 2018 Nigeria Demographic and Health Survey (NDHS 2018).
4. Average Annual Rate of Chang (AARC) is the average change in annual coverage between NDHS 2013 and NDHS 2018. Then, we curved AARC over 5% to 5% and AARC below 0% to 0%.
5. Skilled birth attendance at delivery comprises of a package of services that target mothers and babies. Interventions that target mothers include clean birth practices, labor and delivery management, antibiotics for pre-term premature rupture of the membranes, $MgSO_4$ for the management of eclampsia, and active management of the third stage of labor. Interventions that target children include antibiotics for preterm premature rupture of the membranes, clean birth practices, labor and delivery management, immediate assessment and stimulation of newborn, and neonatal resuscitation.

Table B3. Intervention coverage for each intervention by 2030, disaggregated by wealth quintile

Interventions	S1 Status quo					S2 Uniform scale up					S3 Pro-poor targeted scale-up				
Services coverage (year of achieving 95% coverage)	1st (poorest)	2nd	3rd	4th	5th (wealthiest)	1st (poorest)	2nd	3rd	4th	5th (wealthiest)	1st (poorest)	2nd	3rd	4th	5th (wealthiest)
1. Tetanus toxoid vaccination	68.60	86.92	95(2028)	95(2028)	95(2025)	95(2028)	95(2026)	95(2023)	95(2022)	95(2020)	95(2025)	95(2024)	95(2023)	95(2022)	95(2021)
2. Intermittent preventive treatment of malaria during pregnancy	21.73	19.57	28.55	40.95	43.28	37.97	34.21	49.90	71.56	75.64	64.74	47.25	55.63	64.13	54.28
3. Iron supplementation in pregnancy	30.35	39.15	62.32	73.09	42.80	53.04	68.42	95(2029)	95(2027)	76.86	90.42	94.49	95(2028)	95(2028)	54.28
4. Hypertensive disorder case management in pregnancy	65.46	87.33	86.64	80.25	90.40	95(2029)	95(2026)	95(2023)	95(2022)	95(2020)	95(2025)	95(2024)	95(2023)	95(2023)	95(2021)
5. Malaria case management in pregnancy	95(2025)	95(2025)	95.00	77.22	68.24	95(2022)	95(2022)	95(2023)	95(2027)	95(2028)	95(2021)	95(2021)	95(2023)	95(2028)	85.58
6. Skilled birth assistance at delivery[1]	24.06	42.38	61.89	76.00	84.30	42.05	74.07	95(2029)	95(2025)	95(2021)	71.69	95(2030)	95(2027)	95(2026)	95(2025)
7. Breastfeeding promotion	28.92	35.27	41.80	47.79	63.14	50.59	61.69	73.10	83.59	95(2029)	86.30	85.24	81.50	74.90	79.21
8. Chlorhexidine	9.34	12.93	15.27	16.14	19.60	16.32	22.60	27.02	28.80	35.20	27.82	31.21	30.20	25.71	24.86
9. Complementary feeding-education only	35.38	22.60	44.20	69.55	78.20	61.83	40.59	79.38	95(2026)	95(2022)	95(2030)	56.91	88.94	95(2027)	95(2028)
10. Complementary feeding-supplementary feeding and education	35.38	22.60	44.20	69.55	78.20	61.83	40.59	79.38	95(2026)	95(2022)	95(2030)	56.91	88.94	95(2027)	95(2028)
11. Vitamin A supplementation	60.16	32.99	42.30	69.92	68.00	95(2029)	59.11	75.96	95(2026)	95(2025)	95(2026)	82.79	85.12	95(2028)	86.24
12. Households protected from malaria (ITN+IRS)	95(2024)	95(2024)	92.92	81.19	63.38	95(2021)	95(2022)	95(2024)	95(2025)	95(2028)	95(2020)	95(2021)	95(2023)	95(2026)	79.94
13. Oral rehydration solution	59.98	54.45	47.58	61.43	95(2030)	95(2029)	95(2030)	84.84	95(2029)	95(2023)	95(2026)	95(2027)	94.94	95(2030)	95(2026)
14. Zinc for treatment of diarrhea	46.51	45.26	64.83	69.50	81.17	81.29	79.09	95(2029)	95(2028)	95(2026)	95(2028)	95(2029)	95(2028)	95(2029)	95(2029)
15. Oral antibiotics for pneumonia	16.22	18.50	15.00	18.70	24.30	28.64	32.33	26.94	33.58	43.64	49.28	44.65	30.18	29.94	30.82
16. Artemisinin-based combination treatment of malaria	3.60	9.70	10.13	16.18	30.53	6.47	16.95	17.89	28.42	53.35	11.30	23.41	19.97	25.44	38.29
17. SAM-treatment for severe acute malnutrition	12.81	8.50	8.10	12.60	32.32	22.44	15.22	16.30	22.63	56.60	38.34	21.31	16.30	20.17	40.57
18. MAM-treatment for moderate acute malnutrition	12.81	8.50	8.10	12.60	32.32	22.44	15.22	16.30	22.63	56.60	38.34	21.31	16.30	20.17	40.57

Notes

1. Skilled birth attendance at delivery comprises of a package of services that target mothers and babies. Interventions that target mothers include clean birth practices, labor and delivery management, antibiotics for pre-term premature rupture of the membranes, $MgSO_4$ for the management of eclampsia, and active management of the third stage of labor. Interventions that target children include antibiotics for preterm premature rupture of the membranes, clean birth practices, labor and delivery management, immediate assessment and stimulation of newborn, and neonatal resuscitation.

Section C. Estimation of financial risk protection

To get the private expenditure averted, we first get the total cost (including intervention costs, program costs and health system costs) from LiST, and then multiply the current out-of-pocket payment ratio in Nigeria (77%) to estimate private expenditure averted. LiST estimates the financial resources required to deliver a package of services using several inputs. The determinants for intervention costs are the number of people receiving the intervention and the quantity of resources required to deliver the intervention per person. For each intervention, costed inputs included drugs and consumable supplies (e.g., gloves, syringes), provider time, and number of inpatient days and outpatient visits needed for the effective provision of an intervention. We used the default settings in LiST which were developed based on WHO norms and guidelines. The defaults for drugs and consumable supply prices are extracted from international sources such as the MSH Drug Price Indicator Guide, UNICEF supply catalog, and the Global Price Reporting Mechanism (Spectrum Manual: https://www.livessavedtool.org/).

We define catastrophic health expenditure (CHE) as out-of-pocket payment for health expenditure exceed 10% of household income. We used the income information from the Living Standard Survey, disaggregated by wealth quintile. we estimate the CHE cases averted by following steps

Step 1: Using household income data from 2018 Nigeria integrated survey of agriculture and dividing by average size of household to get the individual income data. We got the lower and upper bound of annual individual income for each quintile (See results in Table C1). We then populated Gamma distribution using GNI per capita (2,157USD) and Gini Index (35.1USD) to get the income distribution within each quintile.

Table C1. Average household income by quintile and Individual equivalents

		Average annual individual income (at 1USD = 305 NGN)	
**Quintile**	**Average household size**	**Lower bound**	**Upper bound**
Q1 (Poorest)	8.1	0.00	1,179.15
Q2	6.6	1,179.16	1,693.57
Q3	5.7	1,693.58	2,244.45
Q4	4.6	2,244.46	3,019.79
Q5 (Wealthiest)	3.3	3,019.80	7,484.51

Step 2: We used the unit cost of each intervention to determine the lower and upper threshold for CHE. If certain intervention's unit cost could lead to CHE, then we estimate proportion of population at risk by using the Gamma distribution.

Step 3: Finally we multiplied the proportion of population at risk of CHE with proportion of population to use the intervention, and the sample size for each quintile, we got number of CHE cases for each quintile.

Section D. Additional tables and Figures

D1. Uniform scale up scenario

Population coverage for target interventions will increase by 5% over and above the status quo every year;

Table D1. Additional under-five and maternal deaths averted through public finance of 18 essential MNCH interventions in Nigeria, disaggregated by income quintile, and intervention.

△ (S2-S1)[1]	Number of under-five deaths averted[2]						Number of maternal deaths averted[2]					
	Quintiles[3]					Total[5]	Quintiles[3]					Total[5]
Interventions	1st (poorest)	2nd	3rd	4th	5th (wealthiest)		1st (poorest)	2nd	3rd	4th	5th (wealthiest)	
TT-Tetanus toxoid vaccination	8,662	6,632	4,125	1,987	879	22,285 (2.1%)	70	59	48	42	17	236 (0.2%)
IPTP-Intermittent preventive treatment of malaria during pregnancy	1,391	1,407	1,430	1,279	912	6,419 (0.6%)	408	349	391	452	299	1,899 (1.7%)
Iron supplementation in pregnancy							1,272	1,565	2,269	2,279	1,537	8,922 (8.0%)
Hypertensive disorder case management in pregnancy							3,799	2,864	2,612	3,007	773	13,055 (11.7%)
Malaria case management in pregnancy							153	121	218	402	277	1,171 (1.1%)
Child birth[4]	27,407	60,354	98,608	93,618	69,902	349,889 (33.3%)	3,654	10,537	19,738	24,431	25,863	84,223 (75.7%)
Breastfeeding promotion	4,670	4,879	4,214	3,620	1,550	18,933 (1.8%)						
Chlorhexidine	1,009	1,343	1,117	1,046	852	5,367 (0.5%)						
Complementary feeding-education only	8,944	6,769	7,739	5,252	1,114	29,818 (2.8%)						
Complementary feeding-supplementary feeding and education	1,990	1,379	1,668	1,171	256	6,464 (0.6%)						
Vitamin A supplementation	22,125	13,834	11,273	8,268	2,567	58,067 (5.5%)						
ITN/IRS-households protected from malaria	8,810	10,412	24,285	21,045	8,332	72,884 (6.9%)	99	457	297	448	457	1,758 (1.6%)
ORS-oral rehydration solution	77,322	80,294	52,641	35,696	5,977	251,930 (24.0%)						
Zinc for treatment of diarrhea	12,964	12,164	9,410	4,828	1,379	40,745 (3.9%)						
Oral antibiotics for pneumonia	30,784	28,854	18,388	14,301	5,557	97,884 (9.3%)						
ACTs-Artemisin compounds for treatment of malaria	5,605	10,716	6,775	5,890	3,076	32,062 (3.1%)						
SAM-treatment for severe acute malnutrition	4,785	3,809	2,726	1,894	791	14,005 (1.3%)						
MAM-treatment for moderate acute malnutrition	10,601	7,956	11,981	9,866	2,333	42,737 (4.1%)						
**All**	**227,069**	**250,802**	**256,380**	**209,761**	**105,477**	**1,049,489**	**9,455**	**15,952**	**25,573**	**31,061**	**29,223**	**111,264**

Notes:

1. Estimates represent the difference between scenarios. For each scenario, additional lives were estimated as the difference between the number or deaths in the index year and the number of deaths in the baseline year (2018). S1 Status quo: Population coverage for target interventions will expand at an annual rate of change (AARC) equal to the trend in the five years between 2013 and 2018; S2 Uniform scale up scenario: Population coverage for target interventions will increase by 5% over and above the status quo every year; S3 Pro-poor targeted scale-up scenario (aggressive scale-up among poorer quintiles): In this scenario, population coverage for target interventions will increase by 10%, 8%, 6%, 4% and 2% over and above the status quo for quintiles 1-5 (from poorest to wealthiest), respectively, every year
2. Number of lives saved and life-years gained were not discounted
3. Quintiles were defined at the beginning of the period (2018). As a result, individuals maintained their relative quintiles throughout the period of interest. The percentage under each quintile indicates the proportion of private expenditure averted for each quintile compared to national total during the period 2019-2030, and disaggregated by intervention.
4. Skilled birth attendance at delivery comprises of a package of services that target mothers and babies. Interventions that target mothers include clean birth practices, labor and delivery management, antibiotics for pre-term premature rupture of the membranes, $MgSO_4$ for the management of eclampsia, and active management of the third stage of labor. Interventions that target children include antibiotics for preterm premature rupture of the membranes, clean birth practices, labor and delivery management, immediate assessment and stimulation of the newborn, and neonatal resuscitation.
5. Includes all 12 years between 2019 and 2030.

Table D2. Additional private expenditure averted from public finance of selected priority MNCH interventions in Nigeria, disaggregated by income quintile, and intervention.

△ (S2-S1)[1]	Private expenditure averted between 2019 to 2030 (in millions of 2018 USD)					
	Quintiles[2]					
**Interventions**	**1st (poorest)**	**2nd**	**3rd**	**4th**	**5th (wealthiest)**	**Total[3]**
TT-Tetanus toxoid vaccination	10.4	7.4	4.1	2.4	0.5	24.8 (1.4%)
IPTP-Intermittent preventive treatment of malaria during pregnancy	3.6	3.0	4.0	5.7	4.3	20.5 (1.2%)
Iron supplementation in pregnancy	7.7	9.2	12.3	11.8	8.1	49.2 (2.8%)
Hypertensive disorder case management in pregnancy	0.4	0.3	0.2	0.3	0.1	1.3 (0.1%)
Malaria case management in pregnancy	0.6	0.4	0.9	2.2	1.7	5.8 (0.3%)
Child birth[4]	75.7	126.5	166.1	138.9	56.6	563.9 (31.9%)
Breastfeeding promotion	14.5	16.3	17.4	19.9	16.1	84.2 (4.8%)
Chlorhexidine	0.2	0.3	0.4	0.4	0.3	1.6 (0.1%)
Complementary feeding-education only	4.6	3.4	6.1	6.8	3.5	24.5 (1.4%)
Complementary feeding-supplementary feeding and education	26.9	19.8	35.7	40.3	20.8	143.5 (8.1%)
Vitamin A supplementation	18.5	12.0	14.7	17.4	12.6	75.3 (4.3%)
ITN/IRS-households protected from malaria	2.8	3.4	12.6	19.1	22.5	60.4 (3.4%)
ORS-oral rehydration solution	49.7	50.7	44.3	48.8	11.6	205.2 (11.6%)
Zinc for treatment of diarrhea	56.2	55.1	62.7	59.3	34.5	267.8 (15.1%)
Oral antibiotics for pneumonia	16.2	15.9	14.8	18.9	17.1	82.9 (4.7%)
ACTs-Artemisin compounds for treatment of malaria	3.9	8.0	8.5	13.3	16.8	50.6 (2.9%)
SAM-treatment for severe acute malnutrition	12.5	10.2	11.3	12.9	10.8	57.6 (3.3%)
MAM-treatment for moderate acute malnutrition	10.2	8.0	9.0	10.9	10.7	48.9 (2.8%)
**All**	**314.8**	**350.0**	**425.1**	**429.5**	**248.7**	**1768.0**

Notes:

1. Estimates represent the difference between scenarios. For each scenario, additional lives were estimated as the difference between the number or deaths in the index year and the number of deaths in the baseline year (2018). S1 Status quo: Population coverage for target interventions will expand at an annual rate of change (AARC) equal to the trend in the five years between 2013 and 2018; S2 Uniform scale up scenario: Population coverage for target interventions will increase by 5% over and above the status quo every year; S3 Pro-poor targeted scale-up scenario (aggressive scale-up among poorer quintiles): In this scenario, population coverage for target interventions will increase by 10%, 8%, 6%, 4% and 2% over and above the status quo for quintiles 1-5 (from poorest to wealthiest), respectively, every year
2. Includes all 12 years between 2019 and 2030.

3. Quintiles were defined at the beginning of the period (2018). As a result, individuals maintained their relative quintiles throughout the period of interest. The percentage under each quintile indicates the proportion of private expenditure averted for each quintile compared to national total during the period 2019-2030, and disaggregated by intervention.
4. Skilled birth attendance at delivery comprises of a package of services that target mothers and babies. Interventions that target mothers include clean birth practices, labor and delivery management, antibiotics for pre-term premature rupture of the membranes, $MgSO_4$ for the management of eclampsia, and active management of the third stage of labor. Interventions that target children include antibiotics for preterm premature rupture of the membranes, clean birth practices, labor and delivery management, immediate assessment and stimulation of the newborn, and neonatal resuscitation.

Table D3. Incremental cost-effectiveness ratios from public finance of MNCH interventions in Nigeria, disaggregated by income quintile, and intervention

△ (S2-S1)[1]	Incremental cost per life saved [2] in 2018 USD						Incremental cost per life year saved [2,4] in 2018 USD					
	Quintiles[3]						Quintiles[3]					
Interventions	1st (poorest)	2nd	3rd	4th	5th (wealthiest)	National[5]	1st (poorest)	2nd	3rd	4th	5th (wealthiest)	National[5]
TT-Tetanus toxoid vaccination	1,862	1,735	1,515	1,814	901	1,717	33.8	31.5	27.6	33.1	16.4	31.2
IPTP-Intermittent preventive treatment of malaria during pregnancy	3,083	2,654	3,407	5,141	5,545	3,850	60.4	51.4	66.4	101.9	109.3	75.4
Iron supplementation in pregnancy	9,418	9,207	8,477	8,081	8,227	8,595	254.2	248.5	228.8	218.1	222.1	232.0
Hypertensive disorder case management in pregnancy	177	162	142	131	125	153	4.8	4.4	3.8	3.5	3.4	4.1
Malaria case management in pregnancy	5,671	5,468	6,763	8,471	9,528	7,727	153.1	147.6	182.5	228.6	257.2	208.6
Child birth[4]	3,799	2,780	2,188	1,834	0	2,024	71.6	53.0	41.9	35.7	0.0	39.2
Breastfeeding promotion	4,828	5,210	6,447	8,582	16,142	6,931	87.5	94.4	116.8	155.5	292.5	125.6
Chlorhexidine	367	353	498	598	634	478	6.6	6.4	9.0	10.8	11.5	8.7
Complementary feeding-education only	808	779	1,231	2,030	4,890	1,279	14.6	14.1	22.3	36.8	88.6	23.2
Complementary feeding-supplementary feeding and education	21,100	22,323	33,338	53,658	126,531	34,592	382.3	404.5	604.1	972.2	2,292.6	626.8
Vitamin A supplementation	1,304	1,357	2,036	3,274	7,663	2,020	23.6	24.6	36.9	59.3	138.8	36.6
ITN/IRS-households protected from malaria	485	487	797	1,386	3,992	1,260	8.8	8.9	14.5	25.3	73.6	23.0
ORS-oral rehydration solution	1,003	984	1,310	2,133	3,038	1,269	18.2	17.8	23.7	38.6	55.0	23.0
Zinc for treatment of diarrhea	6,757	7,057	10,379	19,152	38,998	10,243	122.4	127.9	188.1	347.0	706.6	185.6
Oral antibiotics for pneumonia	818	861	1,256	2,060	4,803	1,321	14.8	15.6	22.8	37.3	87.0	23.9
ACTs-Artemisin compounds for treatment of malaria	1,092	1,170	1,946	3,531	8,519	2,459	19.8	21.2	35.3	64.0	154.4	44.6
SAM-treatment for severe acute malnutrition	4,084	4,153	6,463	10,608	21,195	6,415	74.0	75.3	117.1	192.2	384.0	116.2
MAM-treatment for moderate acute malnutrition	1,505	1,573	1,169	1,729	7,144	1,783	27.3	28.5	21.2	31.3	129.5	32.3
**All interventions**	**2,074**	**2,045**	**2,349**	**2,779**	**2,877**	**2,374**	**38.1**	**37.8**	**43.9**	**52.6**	**56.1**	**44.4**

Notes:

1. Estimates represent the difference between scenarios. For each scenario, additional lives were estimated as the difference between the number or deaths in the index year and the number of deaths in the baseline year (2018). S1 Status quo: Population coverage for target interventions will expand at an annual rate of change (AARC) equal to the trend in the five years between 2013 and 2018; S2 Uniform scale up scenario: Population coverage for target interventions will increase by 5% over and above the status quo every year; S3 Pro-poor targeted scale-up scenario (aggressive scale-up among poorer quintiles): In this scenario, population coverage for target interventions will increase by 10%, 8%, 6%, 4% and 2% over and above the status quo for quintiles 1-5 (from poorest to wealthiest), respectively, every year
2. Includes all 12 years between 2019 and 2030.
3. Quintiles were defined at the beginning of the period (2018). As a result, individuals maintained their relative quintiles throughout the period of interest. The percentage under each quintile indicates the proportion of private expenditure averted for each quintile compared to national total during the period 2019-2030, and disaggregated by intervention.
4. Skilled birth attendance at delivery comprises of a package of services that target mothers and babies. Interventions that target mothers include clean birth practices, labor and delivery management, antibiotics for pre-term premature rupture of the membranes, $MgSO_4$ for the management of eclampsia, and active management of the third stage of labor. Interventions that target children include antibiotics for preterm premature rupture of the membranes, clean birth practices, labor and delivery management, immediate assessment and stimulation of the newborn, and neonatal resuscitation.
5. Includes all 12 years between 2019 and 2030

Table D4. Cost of the public finance of 18 essential MNCH interventions in Nigeria, disaggregated by income quintile, and intervention

Δ (S2-S1)[1]	Intervention cost between 2019 to 2030 (in millions of 2018 USD)						Total health system cost between 2019 to 2030 (in millions of 2018 USD)					
	Quintiles[2]					Total[3]	Quintiles[2]					Total[3]
**Interventions**	**1st (poorest)**	**2nd**	**3rd**	**4th**	**5th (wealthiest)**		**1st (poorest)**	**2nd**	**3rd**	**4th**	**5th (wealthiest)**	
TT-Tetanus toxoid vaccination	13.6	9.7	5.3	3.1	0.7	32.2	16.3	11.6	6.3	3.7	0.8	38.7
IPTP-Intermittent preventive treatment of malaria during pregnancy	4.6	3.9	5.2	7.4	5.6	26.7	5.5	4.7	6.2	8.9	6.7	32.0
Iron supplementation in pregnancy	10.0	12.0	16.0	15.3	10.5	63.9	12.0	14.4	19.2	18.4	12.6	76.7
Hypertensive disorder case management in pregnancy	0.6	0.4	0.3	0.3	0.1	1.7	0.7	0.5	0.4	0.4	0.1	2.0
Malaria case management in pregnancy	0.7	0.6	1.2	2.8	2.2	7.5	0.9	0.7	1.5	3.4	2.6	9.0
Child birth[4]	98.3	164.2	215.8	180.4	73.5	732.3	118.0	197.1	258.9	216.5	88.2	878.7
Breastfeeding promotion	18.8	21.2	22.6	25.9	20.8	109.3	22.5	25.4	27.2	31.1	25.0	131.2
Chlorhexidine	0.3	0.4	0.5	0.5	0.4	2.1	0.4	0.5	0.6	0.6	0.5	2.6
Complementary feeding-education only	6.0	4.4	7.9	8.9	4.5	31.8	7.2	5.3	9.5	10.7	5.4	38.1
Complementary feeding-supplementary feeding and education	35.0	25.7	46.3	52.4	27.0	186.3	42.0	30.8	55.6	62.8	32.4	223.6
Vitamin A supplementation	24.0	15.6	19.1	22.6	16.4	97.8	28.9	18.8	22.9	27.1	19.7	117.3
ITN/IRS-households protected from malaria	3.6	4.4	16.3	24.8	29.2	78.4	4.3	5.3	19.6	29.8	35.1	94.1
ORS-oral rehydration solution	64.6	65.8	57.5	63.4	15.1	266.5	77.5	79.0	69.0	76.1	18.2	319.8
Zinc for treatment of diarrhea	73.0	71.5	81.4	77.1	44.8	347.8	87.6	85.8	97.7	92.5	53.8	417.3
Oral antibiotics for pneumonia	21.0	20.7	19.2	24.5	22.2	107.7	25.2	24.8	23.1	29.5	26.7	129.3
ACTs-Artemisin compounds for treatment of malaria	5.1	10.4	11.0	17.3	21.8	65.7	6.1	12.5	13.2	20.8	26.2	78.8
SAM-treatment for severe acute malnutrition	16.3	13.2	14.7	16.7	14.0	74.9	19.5	15.8	17.6	20.1	16.8	89.8
MAM-treatment for moderate acute malnutrition	13.3	10.4	11.7	14.2	13.9	63.5	16.0	12.5	14.0	17.1	16.7	76.2
**All**	**408.8**	**454.5**	**552.0**	**557.8**	**323.0**	**2,296.1**	**490.6**	**545.5**	**662.4**	**669.3**	**387.6**	**2,755.4**

Notes:

1. Estimates represent the difference between scenarios. For each scenario, additional lives were estimated as the difference between the number or deaths in the index year and the number of deaths in the baseline year (2018). S1 Status quo: Population coverage for target interventions will expand at an annual rate of change (AARC) equal to the trend in the five years between 2013 and 2018; S2 Uniform scale up scenario: Population coverage for target interventions will increase by 5% over and above the status quo every year; S3 Pro-poor targeted scale-up scenario (aggressive scale-up among poorer quintiles): In this scenario, population coverage for target interventions will increase by 10%, 8%, 6%, 4% and 2% over and above the status quo for quintiles 1-5 (from poorest to wealthiest), respectively, every year
2. Includes all 12 years between 2019 and 2030.
3. Quintiles were defined at the beginning of the period (2018). As a result, individuals maintained their relative quintiles throughout the period of interest. The percentage under each quintile indicates the proportion of private expenditure averted for each quintile compared to national total during the period 2019-2030, and disaggregated by intervention.
4. Skilled birth attendance at delivery comprises of a package of services that target mothers and babies. Interventions that target mothers include clean birth practices, labor and delivery management, antibiotics for pre-term premature rupture of the membranes, $MgSO_4$ for the management of eclampsia, and active management of the third stage of labor. Interventions that target children include antibiotics for preterm premature rupture of the membranes, clean birth practices, labor and delivery management, immediate assessment and stimulation of the newborn, and neonatal resuscitation.

D2. Pro-poor targeted scale-up scenario

average annual rates of increase in coverage of essential MNCH interventions: Q1(10%), Q2(8%), Q3(6%), Q4(4%), Q5(2%).

Table D5. Additional under-five and maternal deaths averted through public finance of 18 essential MNCH interventions in Nigeria, disaggregated by income quintile, and intervention.

△ (S3-S1)[1]	Number of under-five deaths averted[2]						Number of maternal deaths averted[2]					
	Quintiles[3]					Total[5]	Quintiles[3]					Total[5]
**Interventions**	**1st (poorest)**	**2nd**	**3rd**	**4th**	**5th (wealthiest)**		**1st (poorest)**	**2nd**	**3rd**	**4th**	**5th (wealthiest)**	
TT-Tetanus toxoid vaccination	12,433	6,473	5,126	1,760	668	26,460 (2.0%)	103	72	51	40	13	279 (0.2%)
IPTP-Intermittent preventive treatment of malaria during pregnancy	3,031	2,352	1,744	1,001	371	8,499 (0.6%)	893	595	477	354	123	2,442 (2.1%)
Iron supplementation in pregnancy							3,108	2,820	2,595	1,971	556	11,050 (9.4%)
Hypertensive disorder case management in pregnancy							5,588	3,484	2,742	2,884	726	15,424 (13.1%)
Malaria case management in pregnancy							198	145	234	353	118	1,048 (0.9%)
Child birth[4]	71,708	101,297	118,999	75,500	43,118	410,622 (30.3%)	11,131	16,660	24,163	18,703	15,251	85,908 (73.0%)
Breastfeeding promotion	11,448	8,872	5,275	2,776	602	28,973 (2.1%)						
Chlorhexidine	2,191	2,155	1,377	810	312	6,845 (0.5%)						
Complementary feeding-education only	21,087	11,864	9,570	4,644	768	47,933 (3.5%)						
Complementary feeding-supplementary feeding and education	4,710	2,489	2,084	1,053	182	10,518 (0.8%)						
Vitamin A supplementation	33,985	24,898	14,044	7,320	1,227	81,474 (6.0%)						
ITN/IRS-households protected from malaria	10,633	11,837	25,784	18,997	3,454	70,705 (5.2%)	174	477	254	450	220	1,575 (1.3%)
ORS-oral rehydration solution	109,873	108,383	64,160	29,769	4,052	316,237 (23.3%)						
Zinc for treatment of diarrhea	23,807	19,179	10,378	4,125	799	58,288 (4.3%)						
Oral antibiotics for pneumonia	73,110	51,319	22,870	11,050	2,031	160,380 (11.8%)						
ACTs-Artemisin compounds for treatment of malaria	13,830	19,471	8,520	4,464	1,287	47,572 (3.5%)						
SAM-treatment for severe acute malnutrition	10,451	6,478	2,693	1,483	312	21,417 (1.6%)						
MAM-treatment for moderate acute malnutrition	25,090	13,928	11,838	8,907	796	60,559 (4.5%)						
**All**	**427,387**	**390,995**	**304,462**	**173,659**	**59,979**	**1,356,482**	**21,195**	**24,253**	**30,516**	**24,755**	**17,007**	**117,726**

Notes:

1. Estimates represent the difference between scenarios. For each scenario, additional lives were estimated as the difference between the number or deaths in the index year and the number of deaths in the baseline year (2018). S1 Status quo: Population coverage for target interventions will expand at an annual rate of change (AARC) equal to the trend in the five years between

2013 and 2018; S2 Uniform scale up scenario: Population coverage for target interventions will increase by 5% over and above the status quo every year; S3 Pro-poor targeted scale-up scenario (aggressive scale-up among poorer quintiles): In this scenario, population coverage for target interventions will increase by 10%, 8%, 6%, 4% and 2% over and above the status quo for quintiles 1-5 (from poorest to wealthiest), respectively, every year

2. Number of lives saved and life-years gained were not discounted
3. Quintiles were defined at the beginning of the period (2018). As a result, individuals maintained their relative quintiles throughout the period of interest. The percentage under each quintile indicates the proportion of private expenditure averted for each quintile compared to national total during the period 2019-2030, and disaggregated by intervention.
4. Skilled birth attendance at delivery comprises of a package of services that target mothers and babies. Interventions that target mothers include clean birth practices, labor and delivery management, antibiotics for pre-term premature rupture of the membranes, $MgSO_4$ for the management of eclampsia, and active management of the third stage of labor. Interventions that target children include antibiotics for preterm premature rupture of the membranes, clean birth practices, labor and delivery management, immediate assessment and stimulation of the newborn, and neonatal resuscitation.
5. Includes all 12 years between 2019 and 2030.

Table D6. Additional private expenditure averted[1] from public finance of selected priority MNCH interventions in Nigeria, disaggregated by income quintile, and intervention.

△ (S3-S1)[1]	Private expenditure averted [2] (in millions of 2018 USD)					
	Quintiles[3]					
Interventions	1st (poorest)	2nd	3rd	4th	5th (wealthiest)	Total
TT-Tetanus toxoid vaccination	14.9	9.0	4.3	2.2	0.4	30.9 (1.4%)
IPTP-Intermittent preventive treatment of malaria during pregnancy	8.7	5.4	5.0	4.4	1.5	25.0 (1.1%)
Iron supplementation in pregnancy	18.9	16.7	14.1	10.2	2.9	62.8 (2.8%)
Hypertensive disorder case management in pregnancy	0.6	0.4	0.2	0.2	0.1	1.5 (0.1%)
Malaria case management in pregnancy	0.7	0.5	1.0	1.9	0.7	4.9 (0.2%)
Child birth[4]	188.1	220.7	188.0	126.6	47.1	770.6 (34.2%)
Breastfeeding promotion	35.6	29.5	21.8	15.3	6.3	108.5 (4.8%)
Chlorhexidine	0.6	0.5	0.4	0.3	0.1	2.0 (0.1%)
Complementary feeding-education only	11.1	6.2	7.6	6.2	2.5	33.6 (1.5%)
Complementary feeding-supplementary feeding and education	64.2	35.8	44.6	36.1	14.9	195.7 (8.7%)
Vitamin A supplementation	28.7	21.9	18.4	15.4	6.1	90.5 (4.0%)
ITN/IRS-households protected from malaria	3.8	4.2	13.5	17.2	9.0	47.7 (2.1%)
ORS-oral rehydration solution	75.7	70.9	54.7	40.3	8.2	249.8 (11.1%)
Zinc for treatment of diarrhea	112.1	91.3	69.4	50.6	19.9	343.2 (15.2%)
Oral antibiotics for pneumonia	40.0	29.0	18.5	14.5	6.1	108.1 (4.8%)
ACTs-Artemisin compounds for treatment of malaria	9.7	14.6	10.6	10.2	6.0	51.2 (2.3%)
SAM-treatment for severe acute malnutrition	27.0	17.5	11.3	10.1	4.5	70.5 (3.1%)
MAM-treatment for moderate acute malnutrition	23.0	14.1	9.0	8.5	4.4	59.0 (2.6%)
**All**	**663.5**	**588.2**	**492.6**	**370.2**	**140.9**	**2255.4**

Notes:

1. Estimates represent the difference between scenarios. For each scenario, additional lives were estimated as the difference between the number or deaths in the index year and the number of deaths in the baseline year (2018). S1 Status quo: Population coverage for target interventions will expand at an annual rate of change (AARC) equal to the trend in the five years between 2013 and 2018; S2 Uniform scale up scenario: Population coverage for target interventions will increase by 5% over and above the status quo every year; S3 Pro-poor targeted scale-up scenario (aggressive scale-up among poorer quintiles): In this scenario, population coverage for target interventions will increase by 10%, 8%, 6%, 4% and 2% over and above the status quo for quintiles 1-5 (from poorest to wealthiest), respectively, every year
2. Includes all 12 years between 2019 and 2030.

3. Quintiles were defined at the beginning of the period (2018). As a result, individuals maintained their relative quintiles throughout the period of interest. The percentage under each quintile indicates the proportion of private expenditure averted for each quintile compared to national total during the period 2019-2030, and disaggregated by intervention.
4. Skilled birth attendance at delivery comprises of a package of services that target mothers and babies. Interventions that target mothers include clean birth practices, labor and delivery management, antibiotics for pre-term premature rupture of the membranes, $MgSO_4$ for the management of eclampsia, and active management of the third stage of labor. Interventions that target children include antibiotics for preterm premature rupture of the membranes, clean birth practices, labor and delivery management, immediate assessment and stimulation of the newborn, and neonatal resuscitation.

Table D7. Incremental cost-effectiveness ratios from public finance of MNCH interventions in Nigeria, disaggregated by income quintile, and intervention

△ (S3-S1)[1]	Incremental cost per life saved [2] in 2018 USD						Incremental cost per life year saved [2,4] in 2018 USD					
	Quintiles[3]						Quintiles[3]					
**Interventions**	**1st (poorest)**	**2nd**	**3rd**	**4th**	**5th (wealthiest)**	**National[5]**	**1st (poorest)**	**2nd**	**3rd**	**4th**	**5th (wealthiest)**	**National[5]**
TT-Tetanus toxoid vaccination	1,851	2,154	1,304	1,891	930	1,798	33.6	39.2	23.7	34.5	17.0	32.7
IPTP-Intermittent preventive treatment of malaria during pregnancy	3,469	2,857	3,490	5,049	4,833	3,566	67.9	55.4	68.0	100.1	95.4	69.7
Iron supplementation in pregnancy	9,462	9,233	8,467	8,100	8,082	8,858	255.4	249.2	228.5	218.6	218.1	239.1
Hypertensive disorder case management in pregnancy	175	161	142	132	125	155	4.7	4.3	3.8	3.6	3.4	4.2
Malaria case management in pregnancy	5,794	5,513	6,777	8,467	9,554	7,298	156.4	148.8	182.9	228.5	257.9	197.0
Child birth[4]	3,539	2,916	2,047	2,094	0	2,419	67.1	55.4	39.3	40.6	0.0	46.5
Breastfeeding promotion	4,844	5,183	6,436	8,601	16,320	5,836	87.8	93.9	116.6	155.8	295.7	105.7
Chlorhexidine	414	397	505	593	615	457	7.5	7.2	9.1	10.8	11.1	8.3
Complementary feeding-education only	818	808	1,246	2,064	5,153	1,091	14.8	14.6	22.6	37.4	93.4	19.8
Complementary feeding-supplementary feeding and education	21,246	22,435	33,368	53,442	127,496	28,991	385.0	406.5	604.6	968.3	2,310.1	525.3
Vitamin A supplementation	1,318	1,371	2,044	3,275	7,711	1,731	23.9	24.8	37.0	59.3	139.7	31.4
ITN/IRS-households protected from malaria	550	527	805	1,377	3,839	1,028	10.0	9.7	14.6	25.1	71.0	18.8
ORS-oral rehydration solution	1,074	1,019	1,328	2,109	3,169	1,231	19.5	18.5	24.1	38.2	57.4	22.3
Zinc for treatment of diarrhea	7,336	7,416	10,428	19,108	38,747	9,176	132.9	134.4	188.9	346.2	702.1	166.3
Oral antibiotics for pneumonia	852	879	1,263	2,047	4,699	1,051	15.4	15.9	22.9	37.1	85.1	19.0
ACTs-Artemisin compounds for treatment of malaria	1,096	1,169	1,935	3,578	7,292	1,676	19.9	21.2	35.1	64.8	132.1	30.4
SAM-treatment for severe acute malnutrition	4,027	4,213	6,556	10,605	22,655	5,128	73.0	76.3	118.8	192.2	410.5	92.9
MAM-treatment for moderate acute malnutrition	1,429	1,578	1,186	1,489	8,650	1,520	25.9	28.6	21.5	27.0	156.7	27.5
**All interventions**	**2,305**	**2,207**	**2,292**	**2,908**	**2,852**	**2,384**	**42.4**	**40.8**	**42.8**	**54.9**	**55.7**	**44.4**

Notes:

1. Estimates represent the difference between scenarios. For each scenario, additional lives were estimated as the difference between the number or deaths in the index year and the number of deaths in the baseline year (2018). S1 Status quo: Population coverage for target interventions will expand at an annual rate of change (AARC) equal to the trend in the five years between 2013 and 2018; S2 Uniform scale up scenario: Population coverage for target interventions will increase by 5% over and above the status quo every year; S3 Pro-poor targeted scale-up scenario (aggressive scale-up among poorer quintiles): In this scenario, population coverage for target interventions will increase by 10%, 8%, 6%, 4% and 2% over and above the status quo for quintiles 1-5 (from poorest to wealthiest), respectively, every year
2. Includes all 12 years between 2019 and 2030.
3. Quintiles were defined at the beginning of the period (2018). As a result, individuals maintained their relative quintiles throughout the period of interest. The percentage under each quintile indicates the proportion of private expenditure averted for each quintile compared to national total during the period 2019-2030, and disaggregated by intervention.
4. Skilled birth attendance at delivery comprises of a package of services that target mothers and babies. Interventions that target mothers include clean birth practices, labor and delivery management, antibiotics for pre-term premature rupture of the membranes, $MgSO_4$ for the management of eclampsia, and active management of the third stage of labor. Interventions that target children include antibiotics for preterm premature rupture of the membranes, clean birth practices, labor and delivery management, immediate assessment and stimulation of the newborn, and neonatal resuscitation.
5. Includes all 12 years between 2019 and 2030

Table D8. Cost of the public finance of 18 essential MNCH interventions in Nigeria, disaggregated by income quintile, and intervention

△ (S2-S1)[1]	Intervention cost between 2019 to 2030 (in millions of 2018 USD)						Total health system cost between 2019 to 2030 (in millions of 2018 USD)					
	Quintiles[2]					Total[3]	Quintiles[2]					Total[3]
**Interventions**	**1st (poorest)**	**2nd**	**3rd**	**4th**	**5th (wealthiest)**		**1st (poorest)**	**2nd**	**3rd**	**4th**	**5th (wealthiest)**	
TT-Tetanus toxoid vaccination	19.3	11.7	5.6	2.8	0.5	40.1	23.2	14.1	6.8	3.4	0.6	48.1
IPTP-Intermittent preventive treatment of malaria during pregnancy	11.3	7.0	6.5	5.7	2.0	32.5	13.6	8.4	7.8	6.8	2.4	39.0
Iron supplementation in pregnancy	24.5	21.7	18.3	13.3	3.7	81.6	29.4	26.0	22.0	16.0	4.5	97.9
Hypertensive disorder case management in pregnancy	0.8	0.5	0.3	0.3	0.1	2.0	1.0	0.6	0.4	0.4	0.1	2.4
Malaria case management in pregnancy	1.0	0.7	1.3	2.5	0.9	6.4	1.1	0.8	1.6	3.0	1.1	7.6
Child birth[4]	244.3	286.7	244.2	164.4	61.2	1,000.7	293.2	344.0	293.0	197.2	73.4	1200.9
Breastfeeding promotion	46.2	38.3	28.3	19.9	8.2	140.9	55.4	46.0	34.0	23.9	9.8	169.1
Chlorhexidine	0.8	0.7	0.6	0.4	0.2	2.6	0.9	0.9	0.7	0.5	0.2	3.1
Complementary feeding-education only	14.4	8.0	9.9	8.0	3.3	43.6	17.3	9.6	11.9	9.6	4.0	52.3
Complementary feeding-supplementary feeding and education	83.4	46.5	57.9	46.9	19.3	254.1	100.1	55.8	69.5	56.3	23.2	304.9
Vitamin A supplementation	37.3	28.4	23.9	20.0	7.9	117.5	44.8	34.1	28.7	24.0	9.5	141.1
ITN/IRS-households protected from malaria	5.0	5.4	17.5	22.3	11.8	61.9	5.9	6.5	21.0	26.8	14.1	74.3
ORS-oral rehydration solution	98.3	92.0	71.0	52.3	10.7	324.4	118.0	110.4	85.2	62.8	12.8	389.3
Zinc for treatment of diarrhea	145.5	118.5	90.2	65.7	25.8	445.7	174.6	142.2	108.2	78.8	31.0	534.9
Oral antibiotics for pneumonia	51.9	37.6	24.1	18.8	8.0	140.4	62.3	45.1	28.9	22.6	9.5	168.5
ACTs-Artemisin compounds for treatment of malaria	12.6	19.0	13.7	13.3	7.8	66.5	15.2	22.8	16.5	16.0	9.4	79.8
SAM-treatment for severe acute malnutrition	35.1	22.7	14.7	13.1	5.9	91.5	42.1	27.3	17.7	15.7	7.1	109.8
MAM-treatment for moderate acute malnutrition	29.9	18.3	11.7	11.0	5.7	76.7	35.9	22.0	14.0	13.3	6.9	92.0
**All**	**861.7**	**763.8**	**639.8**	**480.8**	**183.0**	**2,929.1**	**1034.0**	**916.6**	**767.7**	**577.0**	**219.6**	**3514.9**

Notes:

1. Estimates represent the difference between scenarios. For each scenario, additional lives were estimated as the difference between the number or deaths in the index year and the number of deaths in the baseline year (2018). S1 Status quo: Population coverage for target interventions will expand at an annual rate of change (AARC) equal to the trend in the five years between 2013 and 2018; S2 Uniform scale up scenario: Population coverage for target interventions will increase by 5% over and above the status quo every year; S3 Pro-poor targeted scale-up scenario (aggressive scale-up among poorer quintiles): In this scenario, population coverage for target interventions will increase by 10%, 8%, 6%, 4% and 2% over and above the status quo for quintiles 1-5 (from poorest to wealthiest), respectively, every year
2. Includes all 12 years between 2019 and 2030.
3. Quintiles were defined at the beginning of the period (2018). As a result, individuals maintained their relative quintiles throughout the period of interest. The percentage under each quintile indicates the proportion of private expenditure averted for each quintile compared to national total during the period 2019-2030, and disaggregated by intervention.
4. Skilled birth attendance at delivery comprises of a package of services that target mothers and babies. Interventions that target mothers include clean birth practices, labor and delivery management, antibiotics for pre-term premature rupture of the membranes, $MgSO_4$ for the management of eclampsia, and active management of the third stage of labor. Interventions that target children include antibiotics for preterm premature rupture of the membranes, clean birth practices, labor and delivery management, immediate assessment and stimulation of the newborn, and neonatal resuscitation.

D3. Concentration curves

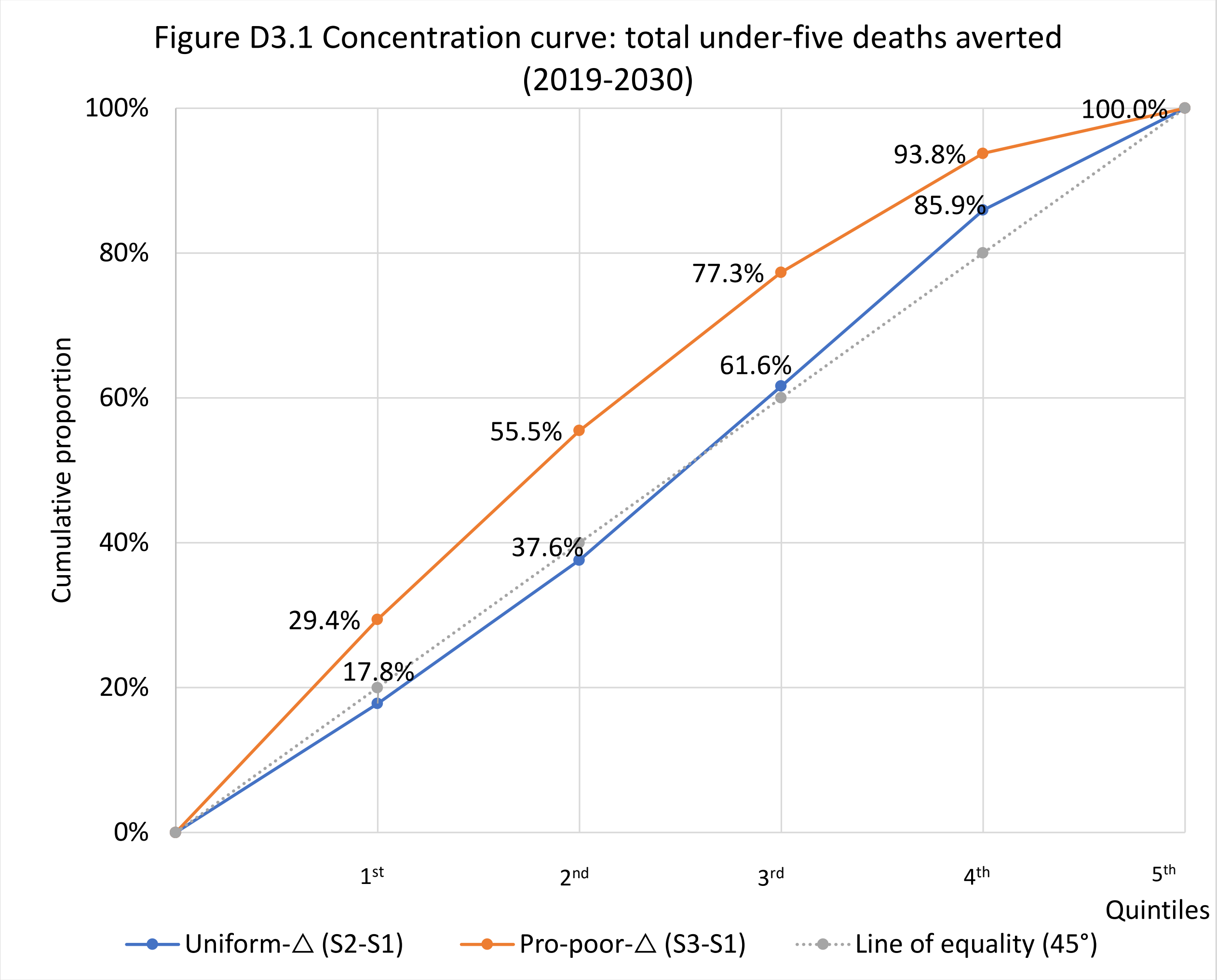

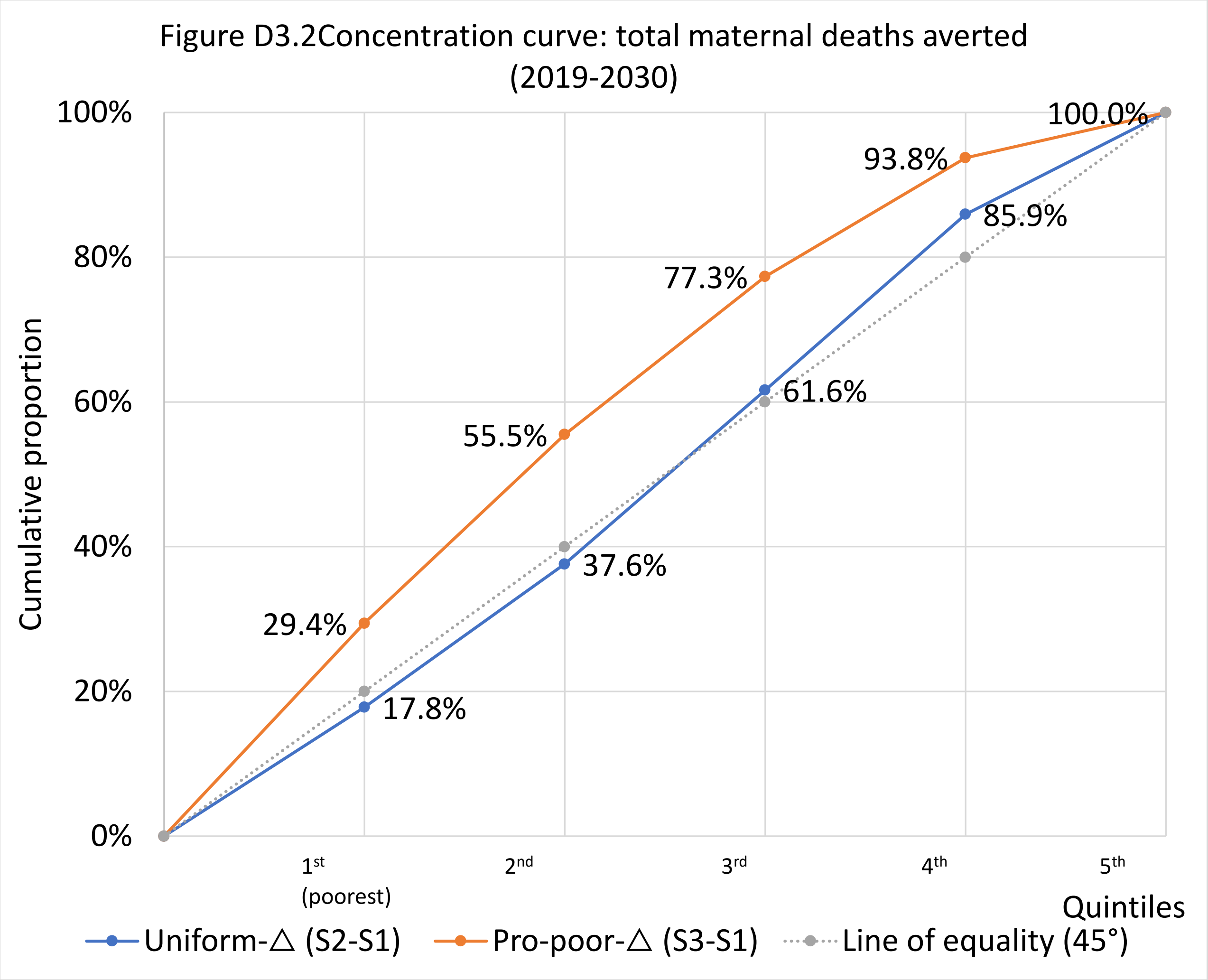
Figure D3.2Concentration curve: total maternal deaths averted (2019-2030)
Cumulative proportion
100%
80%
60%
40%
20%
0%
29.4%
55.5%
77.3%
93.8%
100.0%
17.8%
37.6%
61.6%
85.9%
1st (poorest)
2nd
3rd
4th
5th
Quintiles
Uniform-△ (S2-S1)
Pro-poor-△ (S3-S1)
Line of equality (45°)

Figure D3.3 Concentration curve: private expenditure averted (2019-2030)

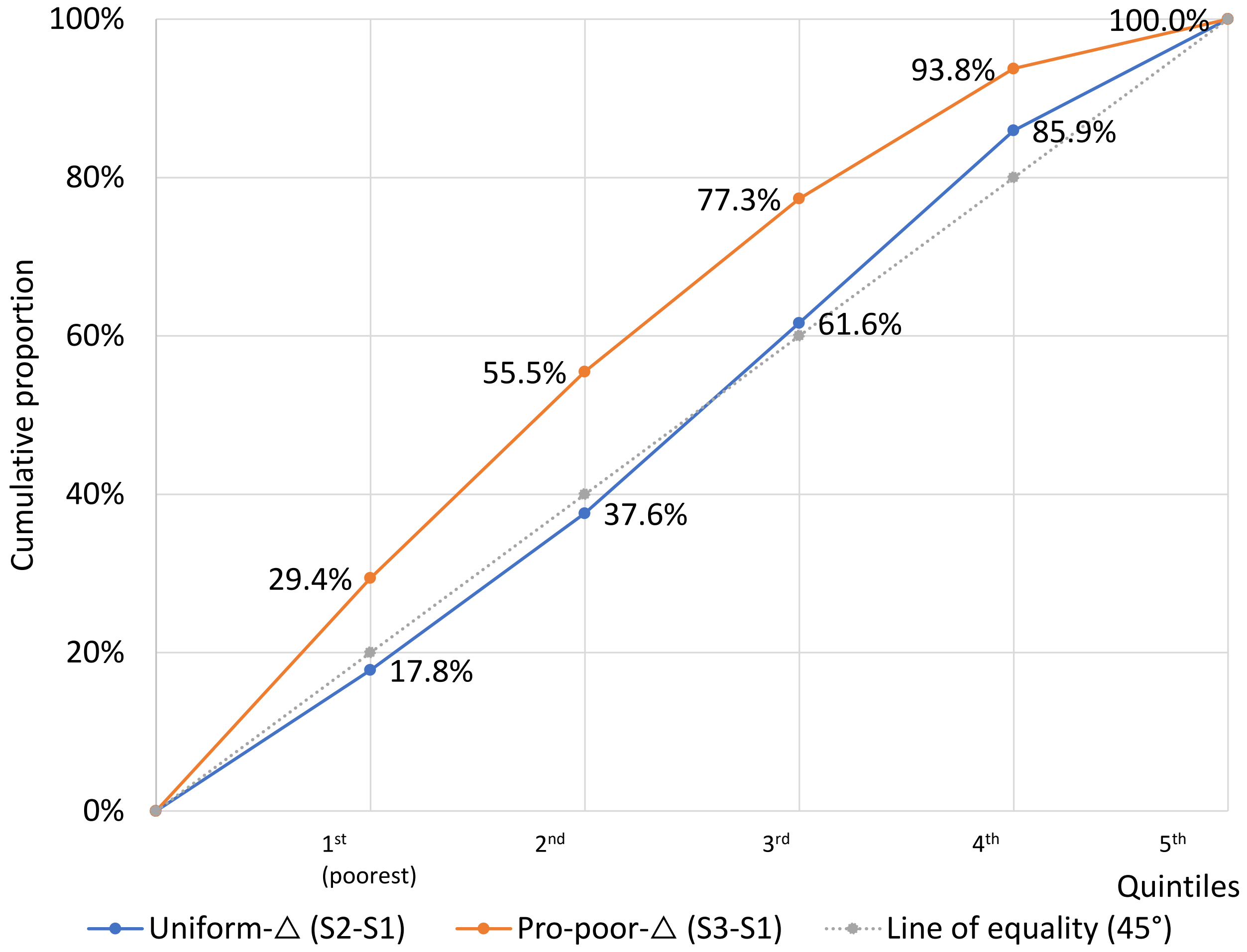

Section E. Sensitivity analysis

Table E1. Summary for sensitivity analysis on TFR, disaggregated by socioeconomic quintiles

	**TFR + 10%**			**TFR - 10%**			**Same TFR for all quintiles**		
	**Lives saved**	**Private expenditure averted**	**ICER**	**Lives saved**	**Private expenditure averted**	**ICER**	**Lives saved**	**Private expenditure averted**	**ICER**
Quintiles									
1st (poorest)	258,097	345.86	1,740	214,905	283.95	2,059	190,821	240.66	1,965
2nd	290,850	384.56	1,717	238,614	315.58	2,061	226,818	300.01	2,061
3rd	310,446	466.37	1,951	256,245	383.83	2,334	267,667	402.98	2,346
4th	264,967	470.26	2,305	216,668	388.27	2,793	227,878	407.17	2,785
5th (wealthiest)	148,360	271.08	2,373	120,959	226.28	2,915	188,748	337.12	2,784
**National**	**1,272,720**	**1938.13**	**1,978**	**1,047,391**	**1597.92**	**2,378**	**1,101,932**	**1687.94**	**2,387**
**Comparing with S5-S2(%)**									
Quintiles									
1st (poorest)	9.1%	9.9%	0.7%	-9.1%	-9.8%	-0.7%	-19.3%	-23.5%	-5.2%
2nd	9.1%	9.9%	0.8%	-10.5%	-9.8%	0.8%	-15.0%	-14.3%	0.8%
3rd	10.1%	9.7%	-0.4%	-9.0%	-9.7%	-0.6%	-5.1%	-5.2%	-0.1%
4th	10.0%	9.5%	-0.5%	-10.0%	-9.6%	0.5%	-5.4%	-5.2%	0.2%
5th (wealthiest)	10.1%	9.0%	-1.0%	-10.2%	-9.0%	1.3%	40.1%	35.6%	-3.3%
**National**	**9.6%**	**9.6%**	**0.0%**	**-9.7%**	**-9.6%**	**0.2%**	**-5.1%**	**-4.5%**	**0.6%**

Commented [WMP1]: TO BE UPDATED:
- ~~Add SA on discount rate~~
- Add 20% as scale up cost in SA -cite other paper use similar approach
- Add SA that S2 increase rate set to 3% vs. 7%

Table E2. Summary for sensitivity analysis on discount rate, disaggregated by socioeconomic quintiles

	Discount rate:3%			Discount rate:5%			Discount rate:10%		
	**Lives saved**	**Private expenditure averted**	**ICER**	**Lives saved**	**Private expenditure averted**	**ICER**	**Lives saved**	**Private expenditure averted**	**ICER**
Quintiles									
1st (poorest)	236,524	412.16	2,716	236,524	492.73	3,247	236,524	766.24	5,049
2nd	266,754	457.91	2,675	266,754	547.18	3,197	266,754	850.16	4,967
3rd	281,953	551.46	3,048	281,953	655.41	3,623	281,953	1,005.36	5,557
4th	240,822	550.84	3,565	240,822	649.94	4,206	240,822	980.44	6,345
5th (wealthiest)	134,700	315.19	3,647	134,700	369.24	4,272	134,700	548.39	6,345
**National**	**1,160,753**	**2,287.55**	**3,071**	**1,160,753**	**2,714.49**	**3,645**	**1,160,753**	**4,150.58**	**5,573**
**Comparing with S5-S2(%)**									
Quintiles	0.0%	30.9%	30.9%	0.0%	56.5%	56.5%	0.0%	143.4%	143.4%
1st (poorest)	0.0%	30.8%	30.8%	0.0%	56.3%	56.3%	0.0%	142.9%	142.9%
2nd	0.0%	29.7%	29.7%	0.0%	54.2%	54.2%	0.0%	136.5%	136.5%
3rd	0.0%	28.3%	28.3%	0.0%	51.3%	51.3%	0.0%	128.3%	128.3%
4th	0.0%	26.7%	26.7%	0.0%	48.5%	48.5%	0.0%	120.5%	120.5%
5th (wealthiest)	0.0%	29.4%	29.4%	0.0%	53.5%	53.5%	0.0%	134.8%	134.8%
**National**	**0.0%**	**30.9%**	**30.9%**	**0.0%**	**56.5%**	**56.5%**	**0.0%**	**143.4%**	**143.4%**

Table E3. Summary for sensitivity analysis on scale up cost, disaggregated by socioeconomic quintiles

	Scale up cost at 10%			Scale up cost at 30%			Scale up cost at 40%		
	**Lives saved**	**Private expenditure averted**	**ICER**	**Lives saved**	**Private expenditure averted**	**ICER**	**Lives saved**	**Private expenditure averted**	**ICER**
Quintiles									
1st (poorest)	236,524	314.78	1,901	236,524	314.78	2,247	236,524	314.78	2,420
2nd	266,754	350.00	1,874	266,754	350.00	2,215	266,754	350.00	2,386
3rd	281,953	425.07	2,154	281,953	425.07	2,545	281,953	425.07	2,741
4th	240,822	429.50	2,548	240,822	429.50	3,011	240,822	429.50	3,243
5th (wealthiest)	134,700	248.68	2,637	134,700	248.68	3,117	134,700	248.68	3,357
**National**	**1,160,753**	**1,768.03**	**2,176**	**1,160,753**	**1,768.03**	**2,572**	**1,160,753**	**1,768.03**	**2,769**
**Comparing with S5-S2(%)**									
Quintiles	0.0%	0.0%	-8.3%	0.0%	0.0%	8.3%	0.0%	0.0%	16.7%
1st (poorest)	0.0%	0.0%	-8.3%	0.0%	0.0%	8.3%	0.0%	0.0%	16.7%
2nd	0.0%	0.0%	-8.3%	0.0%	0.0%	8.3%	0.0%	0.0%	16.7%
3rd	0.0%	0.0%	-8.3%	0.0%	0.0%	8.3%	0.0%	0.0%	16.7%
4th	0.0%	0.0%	-8.3%	0.0%	0.0%	8.3%	0.0%	0.0%	16.7%
5th (wealthiest)	0.0%	0.0%	-8.3%	0.0%	0.0%	8.3%	0.0%	0.0%	16.7%
**National**	**0.0%**	**0.0%**	**-8.3%**	**0.0%**	**0.0%**	**8.3%**	**0.0%**	**0.0%**	**16.7%**

Table E4. Summary for sensitivity analysis on service coverage, disaggregated by socioeconomic quintiles

	Increase 4% service coverage per year			Increase 6% service coverage per year		
	Lives saved	Private expenditure averted	ICER	Lives saved	Private expenditure averted	ICER
Quintiles						
1st (poorest)	222,802	289.12	2,022	297,808	383.80	2,008
2nd	207,175	271.42	2,042	342,338	431.00	1,962
3rd	236,154	343.68	2,268	334,980	492.63	2,292
4th	197,194	369.89	2,923	268,305	477.78	2,775
5th (wealthiest)	121,779	218.79	2,800	140,959	275.21	3,043
**National**	**985,104**	**1492.90**	**2,362**	**1,384,390**	**2060.43**	**2,319**
**Comparing with S5-S2(%)**						
Quintiles						
1st (poorest)	-5.8%	-8.1%	-2.5%	25.9%	21.9%	-3.2%
2nd	-22.3%	-22.5%	-0.2%	28.3%	23.1%	-4.0%
3rd	-16.2%	-19.1%	-3.5%	18.8%	15.9%	-2.5%
4th	-18.1%	-13.9%	5.2%	11.4%	11.2%	-0.2%
5th (wealthiest)	-9.6%	-12.0%	-2.7%	4.6%	10.7%	5.8%
**National**	**-15.1%**	**-15.6%**	**-0.5%**	**19.3%**	**16.5%**	**-2.3%**

Section F. LiST Methodologies

F1 Overview

The Lives Saved Tool (LiST) is computer-based application for modeling the impact of maternal and child health interventions. To estimate maternal, child, or stillbirth outcomes in a projection, LiST models changing coverage for a wide range of maternal and child health interventions over time combined. LiST allows for detailed editing of projection inputs such as: intervention coverage by year, baseline health status, child and maternal mortality rates, stillbirth or abortion information, economic status data, effectiveness of interventions, and the impact of undernutrition on mortality. The key feature of LiST is that it allows one to look at the impact of scaling up coverage of multiple interventions simultaneously, and does not assess only a single intervention or single cause as is the case for many natural history models.

For LiST, the primary inputs are coverage of interventions and the outputs include changes in population-level risk factors or cause-specific mortality. The relationship between changes in inputs (intervention coverage) and one or more outputs is specified in terms of the effectiveness of the intervention for reducing the probability of that outcome. Outcomes of interest include either cause-specific mortality or a risk factor for mortality.

Development of the Lives Saved Tool (LiST) has occurred under the guidance of the Child Health Epidemiology Reference Group (CHERG) of WHO and UNICEF [2]. Although the assumptions used within LiST are drawn from various sources, most of the assumptions about the efficacy and effectiveness of interventions come from a series of journal supplements. National health surveys such as the Demographic and Health Surveys (DHS) and the Multiple Indicator Cluster Surveys (MICS) provide most of the data on current mortality rates, the prevalence of stunting and wasting, and the current coverage of interventions [3].

F2. Structure

The overarching assumption in LiST is that mortality rates and the cause of death structure will not change dynamically, and that any differences will be solely in response to changes in intervention coverage. The model assumes that changes in distal variables such as increases in per capita income or higher levels of maternal education will affect mortality by increasing coverage of interventions or reducing risk factors.

LiST estimates the mortality impact via five age bands: 0 months, 1-5 months, 6-11 months, 12-23 months, and 24-59 months. For each of these age bands, reductions in cause-specific mortality are estimated by applying intervention effectivenesses and affected fractions to intervention coverage changes. The impacts of interventions are calculated separately for eight causes of death in the neonatal period, and nine causes of death in the 1-59 month period. Corrections are then made to this simple equation to correct for the impact of coverage achieved prior to the projection period. The impact of interventions are calculated in groups such that periconceptual, antenatal, and childbirth interventions, sequentially, have the first "opportunity" to prevent mortality, with preventive interventions impacting

mortality next and leaving those that are more curative in nature to reduce the remaining mortality that is not reduced. Then each child who is "saved" is then capable of dying of other causes during the subsequent age period.

LiST also estimates the impact of interventions on maternal mortality. The calculations are very similar to those for child mortality. One difference is that all women aged 15-49 are treated as a single group in the calculations, and interventions act directly on one or more of the nine causes of death (i.e., none affect an intermediate nutrition status). Family planning, although not an intervention in LiST, may also impact maternal mortality by reducing the incidence of abortion, which is a maternal cause of death in the LiST model [4].

A valuable output provided by the LiST model is the attribution of lives saved to changes in coverage of specific interventions and risk factors. When multiple interventions are scaled up that act on the same cause of death, a set of standardized attribution rules is needed. LiST first attributes impact to all preventive interventions (ordered sequentially from periconception, through pregnancy, delivery, followed by the specific age groups described previously), and then attributes impact to the curative interventions, also within this sequential pattern. Thus, if both a preventive and a curative intervention are scaled up simultaneously, the full effect of the change in coverage of the preventive intervention is calculated first and attributed to the preventive intervention. Then any residual deaths averted are calculated and attributed to the curative intervention.

When there are two or more interventions in either preventive or curative categories with an impact on the same cause of death, an additional step is required for the attribution calculation. First, we compute the number of lives saved by applying all preventive interventions. The attribution is based on the proportional impact of the preventive interventions, calculated as the increase in coverage times the effectiveness of the intervention. This same attribution approach is followed if there are three or more preventive interventions and/or curative interventions [4].

The model has several structural features that must be considered in order to appropriately estimate how scaling up coverage of multiple interventions and changing risk factors will impact mortality. First, the effectiveness or efficacy of an intervention must be described in terms of reductions in cause-specific mortality rather than overall mortality. With cause-specific estimates of efficacy, the combined impact of interventions can be computed. Within LiST, the efficacy of an intervention is defined in terms of the reduction of a cause of death or risk factor. The calculation of impact is simple when considering only a single intervention, because the change in coverage times the efficacy of the intervention is applied to the level of cause-specific mortality. By using cause-specific efficacy and applying each intervention to the residual deaths remaining after the previous intervention, LiST ensures that double counting is avoided and the potential impact of multiple interventions is not erroneously inflated [4].

F3. Costing

The objective for developing the LiST costing module was to provide a means for estimating the financial cost of providing a service, while ensuring as much consistency as possible with data already available in LiST, as well as consistency with other methodologies followed by the WHO. Thus an econometric analysis was performed to estimate the contribution of intervention cost components.

The impact estimates of LiST use coverage, effectiveness values and affected fractions to calculate mortality reductions or nutritional status improvements. LiST costing builds on the following related concepts to estimate:

- target population - is the population on which the health intervention is focused, such as pregnant women or children aged <1 month;

- population in need – refers to the percentage of the target population that requires the intervention, such as the percent of pregnant women who need management of pre-eclampsia. For diseases such as diarrhea or malaria where there may be more than one case per year; this can be reflected in a percentage greater than 100%;

- coverage – refers to the effective coverage, i.e. the percentage of the population in need that actually receives the service

- treatment inputs – refers to drugs and supplies, medical personnel time requirements, and number of outpatient visits and inpatient days per case. Treatment inputs can be varied by delivery channel or level of service delivery, in order to reflect variation in drugs and supplies, skilled personnel, and other items that might be required when an intervention is delivered at higher levels of a health system.

- costs per service – calculated based on treatment inputs, and the unit costs for drugs and supplies, provider time, and costs of inpatient days and outpatient visits

In the LiST costing module, the final cost per service is calculated as the multiplication of all of these factors (calculations below are separated into two shorter equations):

- Number of services = Target population * % population in need * coverage.

- Cost per service (for each intervention) = Number of services * unit costs per service.

The intervention cost from List included healthcare provider fees, diagnostic costs, and medication costs but excluded indirect costs such as transportation costs or the opportunity cost of lost employment/wages [5]. For specific cost components and assumptions for each intervention, please refer to OneHealthTool Treatment Assumptions [6].

---

1 National Primary Health Care Development Agency, Nigeria. Available at: https://nphcda.gov.ng/faqs/
2 Walker N, Fischer-Walker C, Bryce J, Bahl R, Cousens C, CHERG Review Groups on Intervention Effects: Standards for CHERG reviews of intervention effects on child survival. International Journal of Epidemiology. 2010, 39 (S 1): i21-i31.
3 Winfrey W, McKinnon R, Stover J. Methods used in the Lives Saved Tool (LiST). BMC Public Health 2011, 11(Suppl 3): S32. http://www.ncbi.nlm.nih.gov/pmc/articles/PMC3231906/
4 Walker N, Tam Y, Friberg I. Overview of the Lives Saved Tool (LiST). BMC Public Health 2013; 13(Suppl 3): S1. http://www.ncbi.nlm.nih.gov/pmc/articles/PMC3847271/.
5 Spectrum Manual. Available at: https://www.livessavedtool.org/ (accessed June 1, 2022)

6 RacheL Sanders. OneHealthTool Treatment Assumptions. Available at: https://avenirhealth.org/download/spectrum/manuals/treatment%20assumptions%202016%201%2010.pdf (Accessed July 1, 2022)
